# Supplementary material for: Using stated-preferences methods to develop a summary metric to determine successful treatment of children with a surgical condition: a study protocol
Source: BMJ Open. 2022 Jun 9;12(6):e062833. doi: 10.1136/bmjopen-2022-062833 (PMC9185585; doi:10.1136/bmjopen-2022-062833)
Supplement: Supplementary data [file bmjopen-2022-062833supp001.pdf]

Welcome and landing page

What is a successful treatment for a child with a surgical condition?
An online survey of people's preferences

Thank you for your interest in taking part in this survey.

Responses to the survey will help us improve the care of children who have a surgical condition.

If you are completing this survey using a tablet or smartphone, we recommend you use landscape mode.

In total, this survey will take about 15-20 minutes to complete. It will take approximately 10 minutes to work through the instructions and warm up questions, and about 5-10 minutes to complete the survey questions themselves.

Please click next to start.

Rivero-Arias O, *et al. BMJ Open* 2022; 12:e062833. doi: 10.1136/bmjopen-2022-062833

Screening questions

Screening question 1 of 3:

Please choose the option which *best* describes you:

- ◯ I am a parent or carer of a child who has been treated for a surgical condition
- ◯ I was treated as a child for a surgical condition
- ◯ I am a healthcare professional caring for children with surgical conditions

Screening question 2 of 3:

What is your age (years):

[ ]

Screening question 3 of 3:

What is your gender?

- ◯ Female
- ◯ Male
- ◯ Other
- ◯ Prefer not to say

Background

Description of the characteristics used in the exercises

We would like you to consider scenarios describing what has happened to an imaginary child after they have been treated for a surgical condition. Before we show you the scenarios, we will take you through the *four characteristics* we will use to describe the scenarios in the exercises. You can read about the characteristics or watch short videos (less than one minute) describing them.

We will ask you some questions about each of these characteristics. **There are no right or wrong answers to these questions,** they are just to help you to get used to the characteristics.

Throughout the survey, the characteristics will be presented in the same order. However, this does not mean that one characteristic is more important than another.

Click next for the first *characteristic.*

Operations (Follow me for a short video)

Most surgical conditions are treated with one or more operations. Some operations that children undergo will be planned at the beginning of that child's treatment, whilst some will be emergencies. How complex an operation is also varies, from minor operations, such as draining an abscess (a pocket of pus under the skin), to more major operations, such as removing sections of intestine (bowel or gut).

In the scenarios you will be shown how many of each of the following types of operation an imaginary child undergoes:

- Planned major operations
- Planned minor operations
- Emergency major operations
- Emergency minor operations

We are only describing **the number of operations** the child had that are **related to their surgical condition**. For example, an operation for appendicitis would not be related.

Please answer the next questions about operations. **There are no right or wrong answers - we are just interested in what you think**.

How many major planned operations do you think are normally needed to treat a child with a broken leg?

No major planned operations	One major planned operation	Two major planned operations	Six major planned operations
○	○	○	○

How many minor planned operations do you think are normally needed to treat a child with a broken leg?

No minor planned operations	One minor planned operation	Two minor planned operations	Six minor planned operations
○	○	○	○

How many <u>major</u> <u>emergency</u> operations do you think are normally needed to treat a child with a <u>broken leg</u>?

No major emergency operations	One major emergency operation	Two major emergency operations	Six major emergency operations

How many <u>minor</u> <u>emergency</u> operations are normally needed to treat a child with a <u>broken leg</u>?

No minor emergency operations	One minor emergency operation	Two minor emergency operations	Six minor emergency operations

Infections treated in hospital (Follow me for a short video)

In some cases, an infection such as a collection of pus within the tummy, may occur shortly after an operation, whilst the child is still in hospital. In other cases, an infection may develop whilst the child is at home, but the infection is still related to their surgical condition and is serious enough for them to need treatment in hospital.

For the scenarios in this survey, you will be shown **the number of infections** the imaginary child had **that were related to their surgical condition and needed treatment in hospital**.

Please answer the next question about infections treated in hospital that will help you to get familar with the terminology used in this survey. **Please remember there are no right or wrong answers - we are just interested in what you think**.

How many <u>infections treated in hospital</u> do you think children usually develop after treatment for a <u>broken leg</u>?

No infections treated in hospital	One infection treated in hospital	Two infections treated in hospital	Six infections treated in hospital

Rivero-Arias O, *et al. BMJ Open* 2022; 12:e062833. doi: 10.1136/bmjopen-2022-062833

Quality of life: The child's quality of life (Follow me for a short video)

In the scenarios we will describe the imaginary child's quality of life as **good, fair, or poor**.

Quality of life is very individual, and what one person thinks of as a good quality of life, someone else may describe as a fair quality of life.

Therefore, we would like you to decide for yourself what you think a good, fair, or poor quality of life for the imaginary child may be.

You might think about whether their condition has had any specific impact on them, for example, resulted in them being incontinent of wee or poo, or need for hospital care or visits. There may also be more broad factors to consider such as how much enjoyment a child gets from day-to-day life, the activities they can do for themselves, and impact on school or home life and family members.

Please read the short story about an imaginary child named Molly and answer the question that follows:

Molly is seven years old and several times a week has accidents where she passes poo in her underwear. Some children at school have started to bully her because of this. As a result, Molly doesn't enjoy school, and often tries to find reasons not to go. Over the last year her development has started to fall behind that of her peers. Molly gets quite stressed and anxious.

Which option below do you think best describes Molly's quality of life?

Good quality of life	Fair quality of life	Poor quality of life
○	○	○

Survival: How long the child survived after their diagnosis (Follow me for a short video)

In each of the scenarios we will describe **how long the imaginary child survived after they were diagnosed** with their surgical condition. This will be presented as one of the following categories:

- Lived more than twenty years, without any expectation that their surgical condition would shorten their life expectancy
- Lived twenty years
- Lived five years
- Lived one year
- Lived six months
- Lived one month

Please answer the next question about survival.

How long do you think children usually survive after being diagnosed with a broken leg?

Lived more than twenty years (normal life expectancy)	Lived twenty years	Lived five years	Lived one year	Lived six months	Lived one month
○	○	○	○	○	○

Practice question paired comparison

Exercise 1

In this exercise, you will be shown a pair of scenarios (**A** and **B**). In each scenario the four *characteristics* describe what has happened to an imaginary child with a surgical condition following their treatment.

We would like you to tell us **whether scenario A or scenario B describes the more successful result of the child's treatment.**

This exercise will be made up of 10 questions each presenting a pair of scenarios. **To make it easier to see the difference between the two scenarios, the characteristics that are the same in both scenarios have a grey background.**

Practice question for Exercise 1

On the next page you will see a practice question.

Which scenario describes the more successful result of the child's treatment?
Click Scenario A or B

**Practice task:**	**Scenario A**	**Scenario B**
**Operations**	Six major planned operations	No major planned operations
	One minor planned operation	One minor planned operation
	Six major emergency operations	No major emergency operations
	One minor emergency operation	One minor emergency operation
**Infections treated in hospital**	One infection treated in hospital	One infection treated in hospital
**Quality of life**	Poor quality of life	Good quality of life
**Survival**	Lived **one month**	Lived more than twenty years (normal life expectancy)
Click Scenario A or B	**Scenario A**	**Scenario B**

Exercise 1 practice question feedback

In the practice question you have just completed, you thought about the characteristics of both scenarios and decided that **Scenario B** was the more successful result of the child's treatment.

Please try to answer all of the following 10 questions by selecting the scenario you think is the **more successful result of the child's treatment**. Try to give each question equal attention.

Remember, there are no right or wrong answers – we are just interested in what you think.

Paired comparisons (participants to complete ten tasks)

Which scenario describes the more successful result of the child's treatment?

Click Scenario A or B

Question #1	Scenario A	Scenario B
Operations	Two major planned operations<br>Six minor planned operations<br>Two major emergency operations<br>Two minor emergency operations	No major planned operations<br>One minor planned operation<br>Six major emergency operations<br>Six minor emergency operations
Infections treated in hospital	Six infections treated in hospital	Six infections treated in hospital
Quality of life	Fair quality of life	Fair quality of life
Survival	Lived more than twenty years (normal life expectancy)	Lived more than twenty years (normal life expectancy)
Click Scenario A or B	Scenario A	Scenario B

NPEU

Nuffield Department of POPULATION HEALTH

FUNDED BY NIHR | National Institute for Health Research

7

Practice question kaizen task

Exercise 2

Each question in **Exercise 2** has three parts:

- In the **first** part you will select between two scenarios (**A** and **B**) as you did in Exercise 1.
- In the **second** part you will see one scenario, again described by the four characteristics. Next to some of the characteristics, you will see an alternative option. You will be asked to **choose three characteristics you think will improve the scenario, starting with the one you think will improve it the most.**
- In the **third** part you will select between two scenarios (**A** and **B**) as you did in Exercise 1.

Practice question for Exercise 2

On the next page you will see a practice question.

UNIVERSITY OF OXFORD

Which scenario describes the more successful result of the child's treatment?

Click Scenario A or B

**Practice question: Part 1.**	**Scenario A**	**Scenario B**
**Operations**	One major planned operation	No major planned operations
	One minor planned operation	No minor planned operations
	One major emergency operation	No major emergency operations
	One minor emergency operation	No minor emergency operations
**Infections treated in hospital**	One infection treated in hospital	No infections treated in hospital
**Quality of life**	Fair quality of life	Fair quality of life
**Survival**	Lived twenty years	Lived **one month**
**Part 1. Click Scenario A or B**	**Scenario A**	**Scenario B**

CSOR

UNIVERSITY OF OXFORD

Which characteristics would you change to improve scenario A?

(Please choose three characteristics from the second column)

**Practice task: Part 2.**	**Scenario A**	**Select the characteristics in the order you would change them**
**Operations**	One major planned operation	
	One minor planned operation	No minor planned operations
	One major emergency operation	No major emergency operations
	One minor emergency operation	No minor emergency operations
**Infections treated in hospital**	One infection treated in hospital	No infections treated in hospital
**Quality of life**	Fair quality of life	
**Survival**	Lived twenty years	
**Part 2.**	**Scenario A**	**Select the characteristics in the order you would change them**

Clear Selection

Which scenario describes the more successful result of the child's treatment?

Click Scenario A or B

**Practice Task:** Part 3.	**Scenario A**	**Scenario B**
**Operations**	One major planned operation	No major planned operations
	No minor planned operations	No minor planned operations
	No major emergency operations	No major emergency operations
	No minor emergency operations	No minor emergency operations
**Infections treated in hospital**	No infections treated in hospital	No infections treated in hospital
**Quality of life**	Fair quality of life	Fair quality of life
**Survival**	Lived twenty years	Lived **one month**
Part 3. Click Scenario A or B	**Scenario A**	**Scenario B**

Exercise 2 practice question feedback

The practice question you have just completed had three parts:

- In the first part you selected **the Scenario A**.
- In the second part you improved three characteristics of **Scenario A**:
  - **First** from one minor planned operation to no minor planned operations
  - **Second** from one major emergency operation to no major emergency operations
  - **Third** from one minor emergency operation to no minor emergency operations
- In the third part you selected the improved **Scenario A.**

Please try to answer all of the following 3 questions. As in the practice question, in the **first part** of each question you need to select a scenario, in the **second part** you need to make three improvements starting with the one you think is most important, and in the **third part** you need to again select a scenario.

Remember, there are no right or wrong answers – we are just interested in what you think.

Kaizen tasks (participants to complete three tasks)

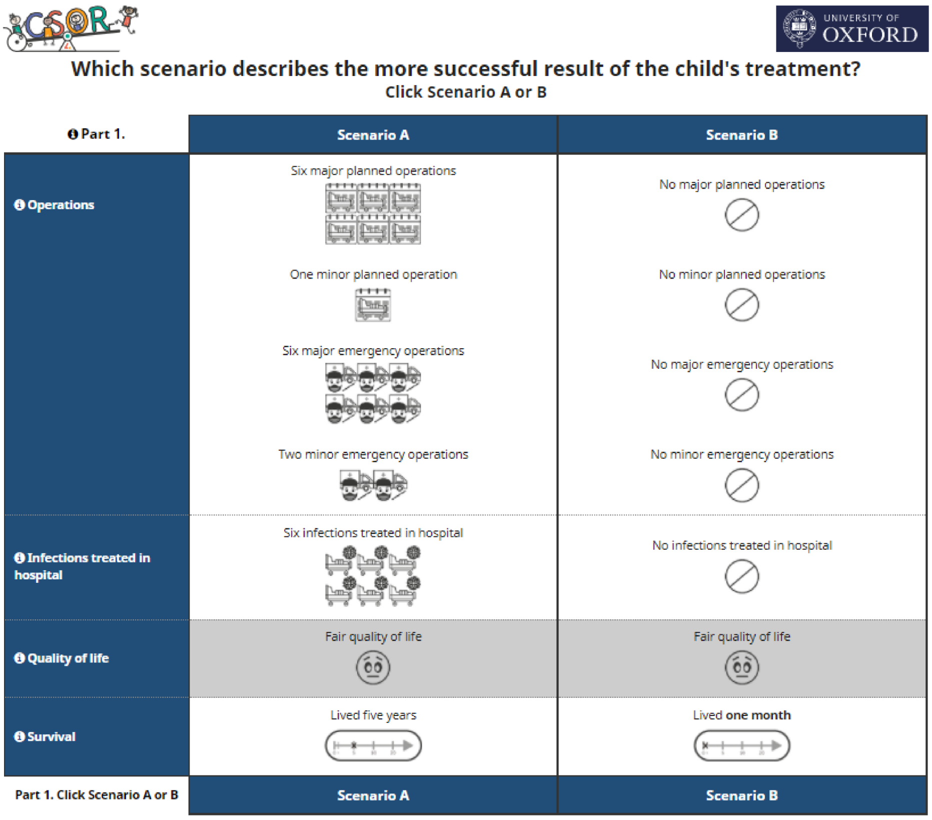

Which scenario describes the more successful result of the child's treatment?
Click Scenario A or B

Part 1.	Scenario A	Scenario B
Operations	Six major planned operations<br>One minor planned operation<br>Six major emergency operations<br>Two minor emergency operations	No major planned operations<br>No minor planned operations<br>No major emergency operations<br>No minor emergency operations
Infections treated in hospital	Six infections treated in hospital	No infections treated in hospital
Quality of life	Fair quality of life	Fair quality of life
Survival	Lived five years	Lived **one month**
Part 1. Click Scenario A or B	Scenario A	Scenario B

Which characteristics would you change to improve scenario A?

(Please choose three characteristics from the second column)

Question 2 Part 2.	Scenario A	Select the characteristics in the order you would change them
Operations	Six major planned operations	
	One minor planned operation	No minor planned operations
	Six major emergency operations	Two major emergency operations
	Two minor emergency operations	
Infections treated in hospital	Six infections treated in hospital	No infections treated in hospital
Quality of life	Fair quality of life	Good quality of life
Survival	Lived five years	
Part 2.	Scenario A	Select the characteristics in the order you would change them

Clear Selection

Which scenario describes the more successful result of the child's treatment?

Click Scenario A or B

Part 3.	Scenario A	Scenario B
Operations	Six major planned operations	No major planned operations
	No minor planned operations	No minor planned operations
	Two major emergency operations	No major emergency operations
	Two minor emergency operations	No minor emergency operations
Infections treated in hospital	No infections treated in hospital	No infections treated in hospital
Quality of life	Good quality of life	Fair quality of life
Survival	Lived five years	Lived **one month**
Part 3. Click Scenario A or B	Scenario A	Scenario B

Debriefing questions

Please tell us what you thought about the two different exercises you just answered

	Exercise 1: Choosing between scenario A and B	Leaning towards Exercise 1	About the same	Leaning towards Exercise 2	Exercise 2: Choosing between scenario A and B and select three improvements
Which exercise did you **prefer to complete**?	○	○	○	○	○
Which exercise did you find **easier to understand**?	○	○	○	○	○
Which exercise did you find **easier to complete**?	○	○	○	○	○

Would you like to comment about the two different exercises you just answered?

Demographics questions (parents and carers)

About you

To help us ensure the results are an accurate description of what a successful treatment is for a child with a surgical condition, it is important that we understand who has completed the survey. Please answer the following questions. It will not be possible to identify you from your responses.

Which country do you usually live in?

- UK
- Ireland
- Australia
- Canada
- New Zealand
- United States of America
- Other:

Please select the surgical condition(s) for which your child has been treated:

- Hirchsprung's disease
- Necrotising enterocolitis
- Gastroschisis
- Oesophageal atresia
- Congenital Diaphragmatic Hernia
- Posterior Urethral Valves
- Other:

Please select which age category your child falls into:

- My child has died
- Less than 1 year old
- 1 to 5 years old
- 6 to 10 years old
- 11 to 18 years old
- More than 18 years old

Please select how you would describe your child's current quality of life:

- ◯ Good quality of life
- ◯ Fair quality of life
- ◯ Poor quality of life
- ◯ Prefer not to answer

Are you:

- ◯ Single
- ◯ Married/Partner
- ◯ Separated
- ◯ Divorced
- ◯ Widowed
- ◯ Prefer not to answer

Please indicate which occupational group the main earner in your household belongs to, or which group fits best:

- ◯ Managers, directors and senior officials
- ◯ Professional/Associate Professional occupations
- ◯ Administrative and secretarial occupations
- ◯ Skilled trades occupations
- ◯ Caring, leisure and other service occupations
- ◯ Sales and customer service occupations
- ◯ Process, plant and machine operatives
- ◯ Elementary occupations
- ◯ Pensioner
- ◯ Unemployed
- ◯ Never employed
- ◯ Prefer not to answer

Do you have a Degree or equivalent professional qualification?

- ◯ Yes
- ◯ No

Have you experienced serious illness in addition to having a child with a surgical condition?

	Yes	No	Prefer not to answer
Yourself	○	○	○
In your family	○	○	○
As a carer for a family member	○	○	○

Demographics questions (individuals treated as child for surgical condition)

About you

To help us ensure the results are an accurate description of what a successful treatment is for a child with a surgical condition, it is important that we understand who has completed the survey. Please answer the following questions. It will not be possible to identify you from your responses.

Which country do you usually live in?

- UK
- Ireland
- Australia
- Canada
- New Zealand
- United States of America
- Other:

Please select the surgical condition(s) for which you were treated as a child:

- Hirchsprung's disease
- Necrotising enterocolitis
- Gastroschisis
- Oesophageal atresia
- Congenital Diaphragmatic Hernia
- Posterior Urethral Valves
- Other:

Please indicate which occupational group the main earner in your household belongs to, or which group fits best:

- Managers, directors and senior officials
- Professional/Associate Professional occupations
- Administrative and secretarial occupations
- Skilled trades occupations
- Caring, leisure and other service occupations
- Sales and customer service occupations
- Process, plant and machine operatives
- Elementary occupations
- Pensioner
- Unemployed
- Never employed
- Prefer not to answer

Do you have a Degree or equivalent professional qualification?

- ○ Yes
- ○ No

Have you experienced serious illness in addition to having a child with a surgical condition?

	Yes	No	Prefer not to answer
Yourself	○	○	○
In your family	○	○	○
As a carer for a family member	○	○	○

Rivero-Arias O, *et al. BMJ Open* 2022; 12:e062833. doi: 10.1136/bmjopen-2022-062833

Demographics questions (healthcare professionals)

About you

To help us ensure the results are an accurate description of what a successful treatment is for a child with a surgical condition, it is important that we understand who has completed the survey. Please answer the following questions. It will not be possible to identify you from your responses.

Which country do you usually live in?

- UK
- Ireland
- Australia
- Canada
- New Zealand
- United States of America
- Other: ______

What is your role/specialism?

- Surgeon
- Anaesthetist
- Paediatrician
- Neonatologist
- Nurse
- Advanced Nurse Practitioner/Specialist Nurse
- Other: ______

For surgeon, anaesthetist, paediatrician and neonatologist

Please select your highest current level of training:

- Consultant or equivalent
- Registrar or equivalent
- Foundation or core trainee
- Prefer not to answer

For nurse, advanced nurse practitioner, specialist nurse

Please select your highest current level of training:

- Consultant
- Modern Matron or Chief Nurse
- Advanced Nurse/Nurse practitioner
- Nursing specialist or Senior Nurse
- Newly qualified nurse
- Prefer not to answer

Have you experienced serious illness in addition to having a child with a surgical condition?

	Yes	No	Prefer not to answer
Yourself	○	○	○
In your family	○	○	○
As a carer for a family member	○	○	○

End of survey and thanks

Thank you for taking part in our survey

If you know other people that you think may be interested in taking part, please tell them about our study so they can register here.

Please do not share your link to the survey with others, as this is unique to you.

If you would like receive email updates on this project, as well as other opportunities to support research in child health, please contact the team at csor@npeu.ox.ac.uk. Your information will be kept securely at the University of Oxford and will not be used for any other purpose or shared with others.

If you would like to talk to someone about how completing the survey has affected you, please get in touch with the CSOR team and we will try to put you in contact with one of the many charities that have been supporting this project.

NPEU

Nuffield Department of POPULATION HEALTH

FUNDED BY NIHR | National Institute for Health Research
