# Supplementary material for: Using stated-preferences methods to develop a summary metric to determine successful treatment of children with a surgical condition: a study protocol
Source: BMJ Open. 2022 Jun 9;12(6):e062833. doi: 10.1136/bmjopen-2022-062833 (PMC9185585; doi:10.1136/bmjopen-2022-062833)
Supplement: Supplementary data [file bmjopen-2022-062833supp002.pdf]

Supplementary material 2: Experimental design for the pair comparison

		Scenario 1							Scenario 2						
**Choice situation**	**Block**	**planned_ major**	**planned_ minor**	**emergency_ major**	**emergency_ minor**	**infection**	**qol**	**survival**	**planned_ major**	**planned_ minor**	**emergency_ major**	**emergency_ minor**	**infection**	**qol**	**survival**
1	3	3	4	3	4	4	3	4	4	4	3	1	3	3	5
2	4	1	3	4	2	4	2	1	2	1	1	2	4	3	1
3	4	1	1	1	3	4	1	1	4	1	4	2	3	1	1
4	5	2	1	3	3	4	3	5	4	1	4	3	3	3	1
5	3	4	2	4	3	4	1	4	4	3	4	1	4	3	3
6	5	4	1	2	4	3	3	3	4	1	4	2	4	3	2
7	3	2	1	2	4	4	3	1	2	1	4	3	3	3	5
8	3	1	1	3	3	4	3	1	3	1	4	1	4	1	1
9	1	4	2	3	1	4	3	5	4	3	2	4	4	2	5
10	2	4	1	3	4	1	1	2	4	3	2	2	1	3	2
11	5	2	4	3	2	2	3	3	2	4	3	4	1	2	5
12	1	3	3	1	4	3	1	1	4	1	2	2	3	1	1
13	4	3	3	4	1	2	3	2	3	4	4	4	2	2	1
14	4	2	2	4	2	4	2	3	4	3	2	2	4	2	1
15	3	2	1	4	1	2	2	5	2	1	4	3	1	3	3
16	3	3	4	4	4	2	3	1	4	1	3	4	2	3	2
17	1	1	4	1	1	3	1	1	2	2	2	1	1	1	1
18	3	2	4	2	2	1	3	4	3	1	2	3	1	3	1
19	3	2	3	3	2	1	3	2	2	4	2	1	4	3	2
20	5	2	3	2	3	2	1	1	4	3	2	1	1	1	4
21	2	2	3	4	4	3	3	2	4	1	4	4	2	3	4
22	2	3	2	2	1	3	3	3	3	3	2	3	2	3	1
23	1	4	1	4	3	1	2	3	4	2	3	3	4	1	3
24	4	3	3	3	3	4	3	5	3	4	3	4	1	3	2
25	5	3	1	2	2	4	1	2	3	1	3	1	2	1	4
26	1	2	4	2	4	3	2	2	3	4	2	2	1	2	3

27	1	3	1	2	2	2	2	5	4	1	4	1	2	2	1
28	5	4	1	2	1	1	2	4	4	2	2	1	2	1	1
29	2	3	2	3	2	4	3	4	4	2	4	2	1	2	4
30	5	3	1	3	4	4	2	1	4	2	1	4	2	2	1
31	2	3	1	2	3	3	1	1	3	4	2	1	2	3	1
32	1	3	1	3	1	4	2	3	3	1	3	4	3	3	2
33	5	2	1	4	3	2	3	4	2	3	4	3	4	1	3
34	4	1	2	2	1	3	3	1	4	2	1	1	4	1	1
35	4	1	4	2	2	2	1	1	4	1	1	2	2	2	1
36	4	2	1	2	4	1	3	1	2	1	3	2	3	1	1
37	2	2	1	2	1	3	1	4	2	4	2	3	2	1	2
38	1	2	1	4	4	3	3	4	4	2	4	4	3	2	2
39	3	3	4	2	3	1	2	5	3	4	3	1	2	2	3
40	2	1	1	1	2	2	3	1	4	1	1	1	4	2	1
41	1	1	2	4	4	4	2	1	3	4	3	3	4	2	1
42	5	3	1	4	4	4	3	3	3	2	3	4	1	3	4
43	2	2	2	3	4	2	3	1	3	1	3	1	2	3	4
44	4	2	3	4	3	3	1	1	3	3	4	2	1	3	1
45	2	3	1	4	3	3	2	1	3	2	4	2	3	1	4

Definitions of attribute levels:
planned_major; planned_minor; emergency_major; emergency_minor coded as:
1 = No operations
2 = One operation
3 = Two operations
4 = Six operations

Infection coded as:
1 = No infection treated in hospital
2 = One infection treated in hospital
3 = Two infections treated in hospital
4 = Six infections treated in hospital

Rivero-Arias O, *et al. BMJ Open* 2022; 12:e062833. doi: 10.1136/bmjopen-2022-062833

Quality of life coded as:
1 = Good quality of life
2 = Fair quality of life
3 = Poor quality of life

Survival coded as:
1 = Lived more than twenty years (normal life expectancy)
2 = Lived twenty years
3 = Lived five years
4 = Lived one year
5 = Lived six months

Rivero-Arias O, *et al. BMJ Open* 2022; 12:e062833. doi: 10.1136/bmjopen-2022-062833
