# Supplementary material for: Using stated-preferences methods to develop a summary metric to determine successful treatment of children with a surgical condition: a study protocol
Source: BMJ Open. 2022 Jun 9;12(6):e062833. doi: 10.1136/bmjopen-2022-062833 (PMC9185585; doi:10.1136/bmjopen-2022-062833)
Supplement: Author's manuscript [file bmjopen-2022-062833.draft_revisions.pdf]

BMJ Open is committed to open peer review. As part of this commitment we make the peer review history of every article we publish publicly available.

When an article is published we post the peer reviewers' comments and the authors' responses online. We also post the versions of the paper that were used during peer review. These are the versions that the peer review comments apply to.

The versions of the paper that follow are the versions that were submitted during the peer review process. They are not the versions of record or the final published versions. They should not be cited or distributed as the published version of this manuscript.

BMJ Open is an open access journal and the full, final, typeset and author-corrected version of record of the manuscript is available on our site with no access controls, subscription charges or pay-per-view fees (http://bmjopen.bmj.com).

If you have any questions on BMJ Open's open peer review process please email info.bmjopen@bmj.com

BMJ Open

Using stated-preferences methods to develop a summary metric to determine successful treatment of children with a surgical condition: a study protocol

Journal:	*BMJ Open*
Manuscript ID	bmjopen-2022-062833
Article Type:	Protocol
Date Submitted by the Author:	14-Mar-2022
Complete List of Authors:	Rivero-Arias, Oliver; University of Oxford, Nuffield Department of Population Health<br>Buckell, John; University of Oxford, Health Economics Research Centre. Nuffield Department of Population Health<br>Allin, Benjamin; University of Oxford, National Perinatal Epidemiology Unit. Nuffield Department of Population Health; Chelsea and Westminster Hospital<br>Craig, Benjamin ; University of South Florida<br>Ayman, Goher; University of Oxford, National Perinatal Epidemiology Unit. Nuffield Department of Population Health<br>Knight, Marian; University of Oxford Nuffield Department of Population Health, National Perinatal Epidemiology Unit<br>Collaborative Group, CSOR; University of Oxford, National Perinatal Epidemiology Unit. Nuffield Department of Population Health.
Keywords:	PAEDIATRIC SURGERY, HEALTH ECONOMICS, HEALTH SERVICES ADMINISTRATION & MANAGEMENT

SCHOLARONE™
Manuscripts

Using stated-preferences methods to develop a summary metric to determine successful treatment of children with a surgical condition: a study protocol

Oliver Rivero-Arias[1], John Buckell[2], Benjamin Allin[1,3], Benjamin M Craig[4], Goher Ayman[1] and Marian Knight[1] on behalf of the CSOR Collaborative Group

1. National Perinatal Epidemiology Unit, Nuffield Department of Population Health, University of Oxford, Oxford, UK
2. Health Economics Research Centre, Nuffield Department of Population Health, University of Oxford, Oxford, UK
3. Chelsea and Westminster Hospital, London, UK
4. Department of Economics, University of South Florida, Tampa, USA

Corresponding author:
Associate Professor Oliver Rivero-Arias
National Perinatal Epidemiology Unit
Nuffield Department of Population Health
Old Road Campus
University of Oxford
OX3 7LF
Email: oliver.rivero@npeu.ox.ac.uk

For peer review only

Abstract

Introduction: Wide variation in the management of key paediatric surgical conditions in the UK has likely resulted in outcomes for some children being worse than they could be. Consequently, it is important to reduce unwarranted variation. However, major barriers to this are the inability to detect differences between observed and expected hospital outcomes based upon the case-mix of the children they have treated, and the inability to detect variation in significant outcomes between hospitals. A stated-preference study has been designed to estimate the value key stakeholders place on different elements of the outcomes for a child with a surgical condition. This study proposes to develop a summary metric to determine what represents successful treatment of children with surgical conditions.

Methods and analysis: Preferences from parents, individuals treated for surgical conditions as infants/children, health care professionals and members of the public will be elicited using paired comparisons and kaizen tasks. A descriptive framework consisting of seven attributes representing types of operations, infections treated in hospital, quality of life and survival was identified. An experimental design has been completed using a D-efficient design with overlap in three attributes and excluding implausible combinations. All participants will be presented with an additional choice task including a palliative scenario that will be used as an anchor. The survey will be administered online. Primary analysis will estimate a mixed multinomial logit model to understand the strengths of preferences associated to attributes for each type of participant and elicitation method. A traffic light system to determine what combination of attributes and levels represent a successful or unsuccessful treatment will be created.

Dissemination: We will disseminate all of our results in peer-review publications and scientific presentations. Findings will be additionally disseminated through relevant charities and support groups, professional organisations and via social media and through a project-specific website.

For peer review only

Strengths and limitations of this study

- To our knowledge, this is the first research study attempting to estimate a summary metric to determine what outcomes represent a successful treatment in children with a surgical condition, facilitating comparisons of hospitals' observed outcomes against their expected outcomes, and comparison of outcomes between hospitals.
- Preference data from two different elicitation techniques, paired comparisons and a novel kaizen task, will be collected contributing to the knowledge-base of the latter for future studies.
- Preference data from different stakeholders relevant to the decision context will be available to estimate the final summary metric.
- Given that children with surgical conditions are relatively few in number, data collection may present challenges, in particular for the identification of parents and health care professionals, which will be mitigated using a thorough recruitment strategy.

For peer review only

1 Introduction

In England and Scotland there are currently 24 Trusts/Health Boards commissioned to provide specialised surgery in children (https://www.england.nhs.uk/commissioning/spec-services/npc-crg/group-e/e02/). Specialised surgery in children includes:

a) Management of rare surgical conditions in children
b) Provision of specified specialised surgical procedures during childhood
c) Surgery in neonates
d) Surgical management or procedures for more common paediatric surgical conditions when a child requires specialist pre-operative, anaesthetic or post-operative care (simple surgical procedures in children with complex medical needs)

Many of the conditions falling under the remit of *specialised surgery in children* commissioning affect only a few hundred children in England and Scotland each year, and what little is known about these children's long-term health and wellbeing suggests that even after treatment, they have significant ongoing healthcare needs [1-7]. Widespread variation in management of children with these conditions currently occurs [1-6], but due to the rarity of the conditions, it has not to date been possible to identify how much of this variation is unwarranted or associated with variation in outcome. To identify unwarranted variation in management and outcome between centres providing *specialised surgery in children* it is necessary to develop mechanisms that will:

1. Collect accurate, unbiased data about children treated in individual centres
2. Combine data from children with different conditions in a way that enables meaningful outcomes analysis
3. Enable adjustment for case-mix factors affecting centres' outcomes

The NIHR funded study "Improving unwarranted variation in outcomes of children's surgery through a new Children's Surgery Outcome Reporting system using routinely available data (CSOR)" investigates whether one unified system is capable of addressing these three issues and therefore reducing unwarranted variation in surgical care. This protocol paper describes the study design of one of the CSOR sub-studies, tackling the second barrier.

For peer review only - http://bmjopen.bmj.com/site/about/guidelines.xhtml

The gold standard approach for comparing outcomes of interest to patients with a specific condition is to use a core outcome set (COS) [8]. Several COS have recently been developed that are relevant to children with a surgical condition [9-12]. Development of these COSs has highlighted that whilst there are attributes of successful treatment that are unique to each individual condition, there are also attributes that are common across conditions. A single outcome measure that combines these common attributes with attributes that accurately reflected the condition specific elements of each COS (e.g. a measure of quality of life) would be able to provide a meaningful assessment of how successful the treatment of a child with any surgical condition has been. The aim of this study is therefore to develop an algorithm to assist in combining these attributes into one summary metric, and therefore determining what constitutes successful treatment of children with a surgical condition. This metric would facilitate combination of data from children with different conditions enabling meaningful outcomes analysis.

Whether a certain combination of common core outcomes across conditions indicates a successful or unsuccessful treatment depends on the value that relevant stakeholders place on the different elements of the core outcomes. Economists employ preference elicitation techniques to determine such values [13]. Stated preference techniques such as discrete choice experiments (DCE) are well-suited to understand the value of potential combinations of core outcomes of paediatric surgery [14]. A DCE is an experiment with choice tasks that elicit preferences indicating how individuals value attributes of alternatives in a decision scenario [14]. The value of a scenario depends on the levels associated to attributes, which are the characteristics of health, treatments or health care services being evaluated [14]. During a DCE, participants are presented with a number of scenarios and are asked to choose their preferred option, trading off among the attributes. DCEs can take different formats, but paired comparisons, where the participant is presented with two or more scenarios and asked to choose one, are most widely used [15]. Other alternatives to paired comparisons exist including best-worst scaling and more recently kaizen tasks [16, 17]. Participants' choices in stated preferences exercises are analysed using discrete choice models, where choices are associated with combinations of attributes and levels to understand participants' preferences (i.e., their relative importance).

For peer review only - http://bmjopen.bmj.com/site/about/guidelines.xhtml

This is a protocol for a stated-preference study designed to estimate the value key stakeholders place on different combinations of health and care outcomes following treatment of a child with a surgical condition. Stakeholders will be presented with a series of paired comparisons and novel kaizen tasks to elicit their preferences. The final product of the collected data will be an algorithm to determine whether the treatment of a child with a surgical condition has been "successful" or "unsuccessful". The use of stakeholder preferences to help in health care decision-making by policymakers has increased considerably across most developed jurisdictions [15]. This has been accompanied by best practice guidance for developing such studies, applied herein [18-21].

For peer review only

1.1 Aims

To understand how parents, guardians and health care professionals caring for children with a surgical condition, individuals treated for surgical conditions as children, and members of the general public value common health and care outcomes following treatment of a child with a surgical condition. Specifically:

A. To estimate the relative importance of key health outcomes following treatment for a surgical condition in childhood for multiple conditions using a paired comparison and a novel kaizen task.
B. To compare preferences between the two sources of preference data and type of participant in the context of children with a surgical condition.
C. To estimate an algorithm using weights derived from the relative importance estimates in A to derive a summary metric that categorises outcomes following surgery in childhood into "successful" or "unsuccessful" outcome.

2 Methods and analysis

2.1 Overview of the framework for the stated-preference study

Figure 1 describes the framework and different phases that will be followed to conduct this study. This protocol describes the following sections: 1) identification and description, 2) experimental design, 3) survey instrument, and 4) statistical analysis.

For peer review only - http://bmjopen.bmj.com/site/about/guidelines.xhtml

2.2 Identification and description

2.2.1 Decision model and descriptive framework

We have followed the most recent guidance on formative research for the identification of attributes and levels for the descriptive system used in this study [21]. Our decision problem explores how to best conceptualise what constitutes a more successful outcome following treatment for a surgical condition in childhood from the values that relevant stakeholders place on key core outcomes across paediatric surgical conditions. Our decision model hypothesises that a successful treatment for a child with a surgical condition can be represented by a combination of characteristics or attributes. In this study, we define attributes as core health outcomes included in available COSs relevant to paediatric surgery. The attributes and associated levels that describe potential outcomes following treatment define our descriptive framework. We used literature reviews, interviews with parents and paediatric surgeons, and group discussions with our Parent Advisory Group (PAG) to determine the descriptive system for the final survey instrument. Our PAG consists of over 100 parents and family of children who have undergone early surgery for conditions including Hirschsprung's disease, gastroschisis, exomphalos, short bowel and necrotising enterocolitis.

An initial list of conceptual attributes were identified through a review of published COS relevant to paediatric surgery. Relevant COS have been developed for children with Hirschsprung's disease, gastroschisis and appendicitis, as well as for children receiving neonatal care in a high-income setting [9-12]. Each of these COS were developed using a combination of literature reviews, an online Delphi process, and consensus meetings, and included in their stakeholder groups, clinicians, allied health professionals, parents, and children or adults previously treated for the target condition. We also conducted focussed discussions with our PAG who had the opportunity to comment on the COS identified in the literature review. The core outcomes identified in the above mentioned conditions are presented in Table 1.

For peer review only

Table 1: Summary of identified core outcomes in neonatal conditions

Category	Core outcome	Included in core outcome set?			
		Hirschsprung's Disease	Gastroschisis	Neonatal care	Appendicitis
Survival	Survival	x	x	x	x
Quality of life	Quality of life	x	x	x	x
	Psychological stress	x			x
	Time away from full activity				x
Adverse events	Unplanned reoperation	x			x
	Number of operations		x		
	Severe gastrointestinal complication		x		
	Retinopathy of prematurity			x	
	Chronic lung disease			x	
	Bowel obstruction				x
	Readmission				x
	Length of hospital stay				x
	Significant infection		x	x	
	Hirschsprung's Associated Enterocolitis	x			
	Necrotising enterocolitis			x	
	Wound infection				x
	Wound complication				x
	Intra-abdominal abscess				x
Condition specific	Faecal incontinence	x			
	Bowel function score	x			
	Voluntary bowel movements	x			
	Urinary incontinence	x			
	Permanent stoma	x			
	Growth		x		
	Time on parenteral nutrition		x		
	Liver disease		x		
	Brain injury on imaging			x	
	Motor/cognitive/visual/hearing ability			x	
	Antibiotic failure				x
	Negative appendicectomy				x
	Recurrent appendicitis				x

For peer review only - http://bmjopen.bmj.com/site/about/guidelines.xhtml

An iterative process to the identification of attributes was followed. In a first step, conducted by the clinical team (BA, MK), overlap in outcomes of importance was identified between the four COS. Each COS also identified outcomes that were relevant only to the condition of interest, and not represented in the other COSs. In a second step, we reviewed these condition-specific outcomes with our PAG in order to determine how best to represent them in the descriptive system. The group concluded that these condition-specific outcomes were highly likely to impact the child's overall quality of life and would therefore be adequately represented through an overarching attribute of quality of life. Three outcome categories including survival, adverse events, and quality of life were common to all four relevant COSs and therefore selected as the initial set of attributes for the descriptive system. Similar outcomes have also been identified as important in other developed paediatric COS [22, 23]. The specific adverse events identified from each COS were also discussed with our PAG and the CSOR paediatric surgeons in a group discussion. The PAG and surgeons agreed that the main adverse events were better summarised as operations, and hospitalisations due to significant infections. Therefore, four core attributes were identified at the end of the second step. A description of each attribute is given next:

1. **Operations**

Most surgical conditions are treated with one or more operations. Some operations that a child might undergo will be planned at the beginning of the child's treatment, whilst some will be emergency, or unplanned operations. The complexity of each operation also varies, from minor operations, such as draining an abscess, to more major operations, such as removing sections of intestine (bowel or gut). For the purpose of the preference study, the number of operations will be presented within four different attributes according to type of operation.

2. **Whether the child has an infection treated in hospital after their operation**

Each of the COSs identified as relevant to paediatric surgery include condition-specific significant infective complications, such as enterocolitis and intra-abdominal abscess. Some also include a more generalised measure of significant infection, *sepsis.* For the purposes of the preference study, the infective complications included in each COS will be represented by the attribute infections treated in hospital, and the levels will define the frequency of infections.

3. **The child's quality of life**

For peer review only - http://bmjopen.bmj.com/site/about/guidelines.xhtml

Each of the COSs included the outcome quality of life, whilst some also specifically included outcomes relating to psychological wellbeing. There are multiple instruments to measure quality of life in children, but they have not been validated in children with a surgical condition. These tools generally describe multiple domains, including social functioning, physical functioning, and psychological wellbeing, with their output generally reported in a continuous manner. However, for the purposes of our descriptive system, quality of life will be categorised as good, fair or poor. The impact of key condition-specific outcomes will be reflected in the child's overall quality of life.

4. How long the child survives after their diagnosis

Although death is relatively uncommon following most surgery in childhood, it is such a significant outcome that all four COSs relating directly to childhood surgery, and the majority of paediatric COSs include it. For the purposes of the descriptive system, this outcome will be presented positively as survival.

The identification of the attribute levels also employed an iterative process. Firstly, we reviewed the epidemiological data available for each of the attributes to guide the range that could be presented to participants. Existing large-scale cohort studies describing the outcomes for children with a range of conditions treated under the specialised surgery in children commissioning, and affecting different regions of the body, modes of presentation, and urgencies of intervention were reviewed [24-37]. A researcher (BA) extracted point estimates and associated measures of uncertainty for each of the attributes. In discussions with two other researchers (OR-A, BC) initial deterministic ordinal levels for each attribute were developed. The selection of levels considered the potential participant cognitive burden and the ability to test appropriate functional forms hypothesis (e.g., linear, quadratic) for quantitative attributes. This initial list of ordinal levels was discussed with paediatric surgeons in a group meeting to ensure their clinical appropriateness and suggest changes to the wording. The language used to describe the levels was refined further following review by members of the PAG. Finally, we conducted three think aloud exercises with different parents who completed a mock choice task of the survey instrument with two of the researchers (OR-A, JB). Parents were given the opportunity during these interviews to comment on the wording used for the attributes and associated levels. The proposed attributes and attribute levels are described in the descriptive framework in Table 2.

For peer review only - http://bmjopen.bmj.com/site/about/guidelines.xhtml

Table 2: Study descriptive framework

**Attributes**	**Attribute Levels**
P*lanned major* operations related to the condition	- No planned major operations<br>- One planned major operation<br>- Two planned major operations<br>- Six planned major operations
P*lanned minor* operations related to the condition	- No planned minor operations<br>- One planned minor operation<br>- Two planned minor operations<br>- Six planned minor operations
Emergency major operations related to the condition	- No emergency major operations<br>- One emergency major operation<br>- Two emergency major operations<br>- Six emergency major operations
Emergency minor operations related to the condition	- No emergency minor operations<br>- One emergency minor operation<br>- Two emergency minor operations<br>- Six emergency minor operations
Infections treated in hospital	- No infections treated in hospital<br>- One infection treated in hospital<br>- Two infections treated in hospital<br>- Six infections treated in hospital
Child's quality of life	- Good quality of life<br>- Fair quality of life<br>- Poor quality of life
How long the child survived after their diagnosis	- More than twenty years, without any expectation that their surgical condition would shorten their life expectancy<br>- Twenty years<br>- Five years<br>- One year<br>- Six months<br>- One month

2.2.2 Elicitation task and format

Two elicitation formats will be used in this study to estimate preferences: a paired comparison and a kaizen task. Each paired comparison will include two scenarios describing combinations of treatment outcomes of a child with a surgical condition without an opt-out option. An example is presented in Supplementary material 1. This type of choice task is the most widely used format in health preference research [15] and has been used previously to elicit preferences for outcomes of surgery [38-40].

Recent work has reported that valuation can be problematic in the context of child health [41, 42]. Eliciting values to inform decision making at the start of life or early childhood requires stakeholders to

For peer review only - http://bmjopen.bmj.com/site/about/guidelines.xhtml

complete tasks from someone else's point of view. There is some evidence that when the tasks in an elicitation exercise refer to someone else's instead of their own preferences, individuals find the exercise strenuous. This is accentuated when the perspective is that of a new-born or a young individual [41]. In a paired comparison, it is easy to understand that choosing between two undesirable outcomes of paediatric surgery could be distressing to stakeholders. Moreover, members of the general public may find it both distressing and unfamiliar as they find it difficult to relate to the decision context [43]. This in turn can affect the preferences elicited in paired comparisons.

In addition to the paired comparisons in this study, we will therefore also administer a series of kaizen tasks to participants (supplementary information 1). Each kaizen task begins with a paired comparison between a single profile and a palliative one (i.e. no operations and no infections, but the child has fair health and dies within one month). Next, the respondent makes three improvements to the single profiles. After these improvements, the respondent completes a final paired comparison between the improved profile and a palliative one. Respondents may find the task of improving a child's health to be more engaging than choosing between two diverse outcomes.

2.3 Experimental design

A preliminary experimental design has been completed for both elicitation exercises using a three-step approach: 1) generation of a design for the paired comparison task and 2) selection of pairs for the first and third part of the kaizen task and 3) selection of profiles for the second part of the kaizen task.

Our preliminary design has employed a *D-efficient* design to identify the combination of pairs to present in the paired comparison [14]. This is a procedure for generating choice tasks (for respondents) in a way that maximises the statistical efficiency of the choice models that will be estimated. Several restrictions were imposed including "overlaps" in three attributes to reduce cognitive burden (similar attribute levels between pairs) and implausible combinations of outcomes. These combinations were identified by the CSOR surgical team and included:

a. Reject scenarios in which quality of life is good, the child survived for less than one year, and the child underwent 20 or more operations

For peer review only - http://bmjopen.bmj.com/site/about/guidelines.xhtml

b. Reject scenarios in which the child underwent any number of minor operations, but no major operations, and survived for less than 20 years

c. Reject scenarios in which the child underwent fewer than six minor operations, no major operations, and quality of life is low

d. Reject scenarios in which quality of life is good, the child survived for less than six months, and the child underwent any major operations

A candidate-set was created including these restrictions and used as the initial candidates in Ngene [44]. We generated a preliminary design with 45 choice tasks divided into five blocks to which participants will be randomly allocated i.e. nine choice tasks in each block. Participants will be randomised to one of these five blocks and the order of the pair in each choice task will also be randomised.

An additional choice task was added to all blocks, which serves as an "anchor". This anchor was a palliative profile defined as having no operations, no infections, fair quality of life and a survival of one month. This anchor will be used to facilitate comparisons between paired comparison and kaizen responses and also when developing the final algorithm for CSOR. The experimental design is presented in supplementary material 2.

The experimental design for the kaizen task was constructed directly from the pairs in supplementary material 2. Given each pair, an initial profile of the kaizen task was constructed from the worst attributes found in the pair. Likewise, the four possible improvements were defined to be the best attributes found in the pair. Therefore, the preference path captured by the kaizen task should agree with the paired comparison response (i.e. the path passes through profile chosen in the paired comparison before passing through the profile not chosen in the paired comparison).

2.4 Survey instrument

The survey will be administered online and will be programmed in Oxford University servers with an open source platform. The survey will consist of an initial participant information and consent form, followed by a general welcome, three screening questions, an introduction to the research question and description of attributes (provided in both written and short video formats). For each attribute,

For peer review only - http://bmjopen.bmj.com/site/about/guidelines.xhtml

respondents will answer warm-up tasks to give their view on the attribute for a hypothetical condition (see supplementary material 1). This will be followed by the preference elicitations starting with the 10 paired comparisons and then the three kaizen tasks. For both tasks, participants will have the opportunity to complete a practice question. At the end of the elicitation tasks, participants will be asked three debriefing questions covering which exercise they found easier to complete, prefer to complete and easier to understand. Finally, a set of demographic questions will be collected including experience with neonatal/childhood surgical conditions, employment status and education qualifications. For health care professionals we will also ask their job title and level of professional experience with neonatal/childhood surgical conditions.

A preliminary mock survey has been completed and is presented in supplementary material 1. In developing the instrument, preliminary testing was undertaken to maximise user understanding as described in section 2.2.1.

2.5 Statistical evaluation

2.5.1 Data collection, recruitment strategy and sampling

The survey instrument will be completed by 1) a sample of parents of children with a surgical condition, 2) participants who had treatment for a surgical condition as a baby or a child, 3) health professionals caring for those who undergo surgery in childhood and 4) members of the general public. Main data collection will commence in October 2021.

Recruitment materials will be distributed via existing contacts e.g. by our PAG, by the project's 'experts by experience', and by the project's healthcare professional team members; registers and mailing lists of support groups, charities and professional groups/bodies; and open advertising through support groups, charities and professional groups/bodies' communication channels e.g. Twitter, Facebook, e-newsletters, and websites. Distribution to health professionals will be via professional bodies. We will also include information on our project website and Twitter account, and advertise via Facebook to share the opportunity as widely as possible. These recruitment strategies have been successfully used in previous quantitative and qualitative studies [7, 9, 10, 45, 46].

For peer review only - http://bmjopen.bmj.com/site/about/guidelines.xhtml

We will use an online panel company to invite adult members of the general public to complete the survey. We will use quotas and a target recruitment strategy to ensure the sample is representative of the UK general population in terms of gender, age, social grade and nation.

In this study we aim to collect 200 responses from parents and individuals with a surgical condition and 200 responses from health care professionals (total of 400) over a three-month period. We are also aiming to collect 400 responses from members of the general public. This is based on typical sample sizes in the health literature and on simple minimum sample size principles [47]. Since there are no prior studies, it is not possible to obtain prior values for accurate power calculations. However, we will conduct a pilot study of around 80 individuals to assess whether our choice models will have sufficient power to detect significant differences.

The pilot study will also be used to assess the feasibility of the survey instrument in terms of finding programming errors and the process of data capture. Participants will all be recruited from the UK.

2.5.2 Data analysis

In the primary analyses, we will estimate a mixed multinomial logit model with individual-clustered standard errors. Unlike the conditional logit, the mixed specification relaxes the assumption of preference homogeneity by allowing individual-specific random parameters [48]. In the secondary analyses, we will explore preference heterogeneity further by estimating heteroscedastic and latent-class logit models. Although the secondary analysis of preference heterogeneity is more descriptive, we hypothesise that some respondents follow lexicographic processes and that respondents who complete the survey or task more quickly may exhibit greater variance in their responses than respondents who take more time to complete the survey or task. Other behaviours (e.g., task sequence, object position) may also be related to stochastic processes.

In the primary analysis, we will test the following hypotheses:

1. Coefficients are logically consistent and with expected directions.
2. The coefficient for the anchor scenario is negative.
3. Survival and quality of life matter significantly more than the remaining attributes

For peer review only - http://bmjopen.bmj.com/site/about/guidelines.xhtml

4. Survival matters more if child lives in good quality of life and survival matters less if child lives in poor quality of life.

5. Reductions in major planned or emergency operations matter more than reductions in minor or emergency operations.

6. We will observe differences among preferences from the different types of participants but not necessarily between elicitation tasks.

Each type of participant (parents, people who were treated for a surgical condition, health care professionals and members of the general public) will have completed the paired comparison and kaizen tasks. Therefore, eight sets of preference data will be available for analysis. We will compare preferences between types of respondent within each elicitation tasks using two approaches. First, latent scale coefficients of the attribute levels will be rescaled using the coefficient for the anchor scenario and predicted choice probabilities for types of respondent compared using mean square and absolute errors. Second, we will examine the relative attribute importance (RAI) scores by attribute. This involves estimating the range of level combinations for each attribute and applying an attribute-based normalisation to enable comparisons [49]. A similar approach will be used to compare preference evidence between elicitation tasks.

The decision about which coefficients to use in the final algorithm for CSOR will be made by the CSOR Co-investigator Group based on the results of the primary and secondary analyses, feedback from participants, and the face validity of the coefficients obtained.

For the final selected model with rescaled coefficients, we will predict the distribution of utilities of all possible combinations of attribute and levels. This distribution will be used to determine the likelihood of a combination to be considered successful or unsuccessful using a traffic light system: green area (high chance to be successful), amber area (uncertainty about success) and red area (not successful). The external validity of this algorithm will be evaluated in a separate study.

This study is expected to be completed by December 2022.

For peer review only - http://bmjopen.bmj.com/site/about/guidelines.xhtml

2.6 Ethics and dissemination

2.6.1 Ethical considerations

Ethics approval to conduct this study has been obtained from the Medical Sciences Inter-Divisional Research Ethics Committee (IDREC) at the University of Oxford (R59631/RE001-05). Informed consent will be obtained for all participants at the start of the survey.

2.6.2 Dissemination

We will disseminate all of our results in peer-review publications and scientific presentations. A lay summary of the findings will be created using our PAG and circulated to parent support networks and the British Association of Paediatric Surgeons, via social media and on the project website.

2.7 Patient and Public Involvement

Please see section 2.2.

For peer review only

For peer review only - http://bmjopen.bmj.com/site/about/guidelines.xhtml

Footnotes

Author's contributions: OR-A and MK developed the original idea for the study; OR-A, JB, BC, BA and MK were responsible for the study design. OR-A, JB, BA and GA conducted the formative research component informing the development of attributes and levels for the descriptive system. JB, BC and OR-A carried out the experimental design. OR-A and BC implemented the online survey instrument. OR-A drafted the manuscript; all authors revised and approved the final manuscript. All members of the CSOR Collaborative Group participated in the overall programme design and the development and testing of the survey instrument.

Funding statement: This study is funded by the National Institute of Health Research (NIHR) Health Services and Delivery Research (HS&DR) Programme (project reference NIHR 127844). MK is an NIHR Senior Investigator.

Competing interest statement: None

Word count: 4,022

Acknowledgments: We are grateful to our Parental Advisory Group and the paediatric surgeons for their valuable contributions and help with the descriptive system. We would like to thank the participants of our think-aloud exercise, whose suggestions refined the final descriptive system and improved the online survey instrument. We are indebted to Lisa Hinton and Karolina Kuberska for their thoughtful comments on earlier versions of the survey instrument.

CSOR Collaborative Group

Alexander Macdonald (Chelsea & Westminster Hospital), Anna May Long (Cambridge University Hospitals NHS Foundation Trust), Gregor Walker (Royal Hospital for Children, Glasgow), Kokila Lakhoo (Oxford University Hospitals NHS Foundation Trust, University of Oxford), Nigel Hall (Southampton General Hospital and University of Southampton), Nick Lansdale (Royal Manchester Children's Hospital), Simon Kenny (Alder Hey Children's Hospital NHS Foundation Trust), Stefano Giuliani (Great Ormond Street Hospital), Timothy Bradnock (Royal Hospital for Children, Glasgow),

For peer review only - http://bmjopen.bmj.com/site/about/guidelines.xhtml

Joanne Shepherd (Patient Representative), Karolina Kuberska (The Healthcare Improvement Studies Institute (THIS Institute), University of Cambridge), Lisa Hinton (The Healthcare Improvement Studies Institute (THIS Institute), University of Cambridge), University of Cambridge), Goher Ayman (National Perinatal Epidemiology Unit, University of Oxford), Benjamin Allin (National Perinatal Epidemiology Unit, University of Oxford & Chelsea and Westminster Hospital), Charles Opondo (National Perinatal Epidemiology Unit, University of Oxford), Crispin Jenkinson (Health Services Research Unit, University of Oxford), John Buckell (Health Economics Research Centre, University of Oxford), Marian Knight (National Perinatal Epidemiology Unit, University of Oxford), Rema Ramakrishnan (National Perinatal Epidemiology Unit, University of Oxford), Oliver Rivero-Arias (National Perinatal Epidemiology Unit, University of Oxford)

For peer review only

3 References

1. Bradnock, T.J., et al., *Hirschsprung's disease in the UK and Ireland: incidence and anomalies.* Arch Dis Child, 2017. **102**(8): p. 722-727.
2. Owen, A., et al., *Gastroschisis: a national cohort study to describe contemporary surgical strategies and outcomes.* J Pediatr Surg, 2010. **45**(9): p. 1808-16.
3. Allin, B., et al., *A UK wide cohort study describing management and outcomes for infants with surgical Necrotising Enterocolitis.* Sci Rep, 2017. **7**: p. 41149.
4. Brownlee, E., et al., *Current epidemiology and antenatal presentation of posterior urethral valves: Outcome of BAPS CASS National Audit.* J Pediatr Surg, 2019. **54**(2): p. 318-321.
5. Long, A.M., et al., *Early population-based outcomes of infants born with congenital diaphragmatic hernia.* Arch Dis Child Fetal Neonatal Ed, 2018. **103**(6): p. F517-f522.
6. Burge, D.M., et al., *Contemporary management and outcomes for infants born with oesophageal atresia.* Br J Surg, 2013. **100**(4): p. 515-21.
7. Allin, B.S.R., et al., *Outcomes at five to eight years of age for children with Hirschsprung's disease.* Arch Dis Child, 2020.
8. Williamson, P.R., et al., *The COMET Handbook: version 1.0.* Trials, 2017. **18**(Suppl 3): p. 280.
9. Sherratt, F.C., et al., *Core outcome set for uncomplicated acute appendicitis in children and young people.* Br J Surg, 2020. **107**(8): p. 1013-1022.
10. Allin, B.S.R., et al., *NETS(1HD) study: development of a Hirschsprung's disease core outcome set.* Arch Dis Child, 2017. **102**(12): p. 1143-1151.
11. Allin, B.S.R., et al., *Development of a gastroschisis core outcome set.* Arch Dis Child Fetal Neonatal Ed, 2019. **104**(1): p. F76-F82.
12. Webbe, J.W.H., et al., *Core outcomes in neonatology: development of a core outcome set for neonatal research.* Arch Dis Child Fetal Neonatal Ed, 2020. **105**(4): p. 425-431.
13. Weernink, M.G.M., et al., *A Systematic Review to Identify the Use of Preference Elicitation Methods in Healthcare Decision Making.* Pharmaceutical Medicine, 2014. **28**(4): p. 175-185.
14. Hensher, D.A., J.M. Rose, and W.H. Greene, *Applied Choice Analysis*. 2nd edition ed. 2015, Cambridge: Cambridge University Press.
15. Soekhai, V., et al., *Discrete Choice Experiments in Health Economics: Past, Present and Future.* Pharmacoeconomics, 2019. **37**(2): p. 201-226.
16. Louviere, J.J., T.N. Flynn, and A.A.J. Marley, *Best-worst scaling: methods and applications*. 2015, Cambridge: Cambridge University Press.
17. Craig, B.M., K. Rand, and J.D. Hartman, *Preference Paths and Their Kaizen Tasks for Small Samples.* Patient, 2021.
18. Bridges, J.F., et al., *Conjoint analysis applications in health--a checklist: a report of the ISPOR Good Research Practices for Conjoint Analysis Task Force.* Value Health, 2011. **14**(4): p. 403-13.
19. Hauber, A.B., et al., *Statistical Methods for the Analysis of Discrete Choice Experiments: A Report of the ISPOR Conjoint Analysis Good Research Practices Task Force.* Value Health, 2016. **19**(4): p. 300-15.
20. Lancsar, E., D.G. Fiebig, and A.R. Hole, *Discrete Choice Experiments: A Guide to Model Specification, Estimation and Software.* Pharmacoeconomics, 2017. **35**(7): p. 697-716.
21. Hollin, I.L., et al., *Reporting Formative Qualitative Research to Support the Development of Quantitative Preference Study Protocols and Corresponding Survey Instruments: Guidelines for Authors and Reviewers.* Patient, 2020. **13**(1): p. 121-136.
22. Sinha, I.P., et al., *Development of a core outcome set for clinical trials in childhood asthma: a survey of clinicians, parents, and young people.* Trials, 2012. **13**: p. 103.
23. Fink, E.L., et al., *Development of a core outcome set for pediatric critical care outcomes research.* Contemp Clin Trials, 2020. **91**: p. 105968.
24. Allin, B., et al., *Outcomes at one-year post anastomosis from a national cohort of infants with oesophageal atresia.* PLoS One, 2014. **9**(8): p. e106149.
25. Allin, B., et al., *A UK wide cohort study describing management and outcomes for infants with surgical Necrotising Enterocolitis.* Sci Rep, 2017. **7**: p. 41149.
26. Allin, B.S.R., et al., *One-year outcomes following surgery for necrotising enterocolitis: a UK-wide cohort study.* Arch Dis Child Fetal Neonatal Ed, 2018. **103**(5): p. F461-F466.
27. Allin, B.S.R., et al., *Outcomes at five to eight years of age for children with Hirschsprung's disease.* Arch Dis Child, 2020: p. archdischild-2020-320310.

For peer review only - http://bmjopen.bmj.com/site/about/guidelines.xhtml

28. Allin, B.S.R., et al., *Management of Gastroschisis: Results From the NETS2G Study, a Joint British, Irish, and Canadian Prospective Cohort Study of 1268 Infants.* Ann Surg, 2021. **273**(6): p. 1207-1214.
29. Abellan-Perpinan, J.M., H. Bleichrodt, and J.L. Pinto-Prades, *The predictive validity of prospect theory versus expected utility in health utility measurement.* J Health Econ, 2009. **28**(6): p. 1039-47.
30. Long, A.M., et al., *Early population-based outcomes of infants born with congenital diaphragmatic hernia.* Arch Dis Child Fetal Neonatal Ed, 2018. **103**(6): p. F517-F522.
31. Long, A.M., et al., *Oesophageal atresia with no distal tracheoesophageal fistula: Management and outcomes from a population-based cohort.* J Pediatr Surg, 2017. **52**(2): p. 226-230.
32. Long, A.M., et al., *One-year outcomes of infants born with congenital diaphragmatic hernia: a national population cohort study.* Arch Dis Child Fetal Neonatal Ed, 2019. **104**(6): p. F643-F647.
33. Bradnock, T.J., et al., *Hirschsprung's disease in the UK and Ireland: incidence and anomalies.* Arch Dis Child, 2017. **102**(8): p. 722-727.
34. Bradnock, T.J., et al., *Gastroschisis: one year outcomes from national cohort study.* BMJ, 2011. **343**: p. d6749.
35. Brownlee, E., et al., *Current epidemiology and antenatal presentation of posterior urethral valves: Outcome of BAPS CASS National Audit.* J Pediatr Surg, 2019. **54**(2): p. 318-321.
36. Wragg, R., et al., *The postnatal management of boys in a national cohort of bladder outlet obstruction.* J Pediatr Surg, 2019. **54**(2): p. 313-317.
37. Wang, Y., et al., *One-year outcomes for congenital diaphragmatic hernia.* BJS Open, 2019. **3**(3): p. 305-313.
38. Ahmadi, S., et al., *Patient Preferences Around Extent of Surgery in Low-Risk Thyroid Cancer: A Discrete Choice Experiment.* Thyroid, 2020. **30**(7): p. 1044-1052.
39. van Dijk, J.D., et al., *An Empirical Comparison of Discrete Choice Experiment and Best-Worst Scaling to Estimate Stakeholders' Risk Tolerance for Hip Replacement Surgery.* Value Health, 2016. **19**(4): p. 316-22.
40. Szawlowski, S., et al., *How do surgeons' trade-off between patient outcomes and risk of complications in total knee arthroplasty? a discrete choice experiment in Australia.* BMJ Open, 2019. **9**(7): p. e029406.
41. Rowen, D., et al., *Review of Valuation Methods of Preference-Based Measures of Health for Economic Evaluation in Child and Adolescent Populations: Where are We Now and Where are We Going?* Pharmacoeconomics, 2020. **38**(4): p. 325-340.
42. Powell, P.A., et al., *Valuing child and adolescent health: a qualitative study on different perspectives and priorities taken by the adult general public.* Health Qual Life Outcomes, 2021. **19**(1): p. 222.
43. Craig, B.M., et al., *Valuation of Child Health-Related Quality of Life in the United States.* Health Econ, 2016. **25**(6): p. 768-77.
44. ChoiceMetrics, *Ngene 1.2 User Manual & Reference Guide*. 2018, Australia.
45. Allin, B.S.R., et al., *Development of a gastroschisis core outcome set.* Arch Dis Child Fetal Neonatal Ed, 2019. **104**(1): p. F76-f82.
46. Long, A.-M., *Short and long-term outcomes of children born with abdominal wall defects* in *National Perinatal Epidemiology Unit*. 2017, University of Oxford.
47. Reed Johnson, F., et al., *Constructing experimental designs for discrete-choice experiments: report of the ISPOR Conjoint Analysis Experimental Design Good Research Practices Task Force.* Value Health, 2013. **16**(1): p. 3-13.
48. Train, K., *Discrete choice methods with simulation*. 2nd ed. ed. 2009, Cambridge: New York, NY : Cambridge University Press.
49. Gonzalez, J.M., *A Guide to Measuring and Interpreting Attribute Importance.* Patient, 2019. **12**(3): p. 287-295.

For peer review only - http://bmjopen.bmj.com/site/about/guidelines.xhtml

Figure captions

Figure 1: A framework for stated-preference studies

For peer review only

For peer review only - http://bmjopen.bmj.com/site/about/guidelines.xhtml

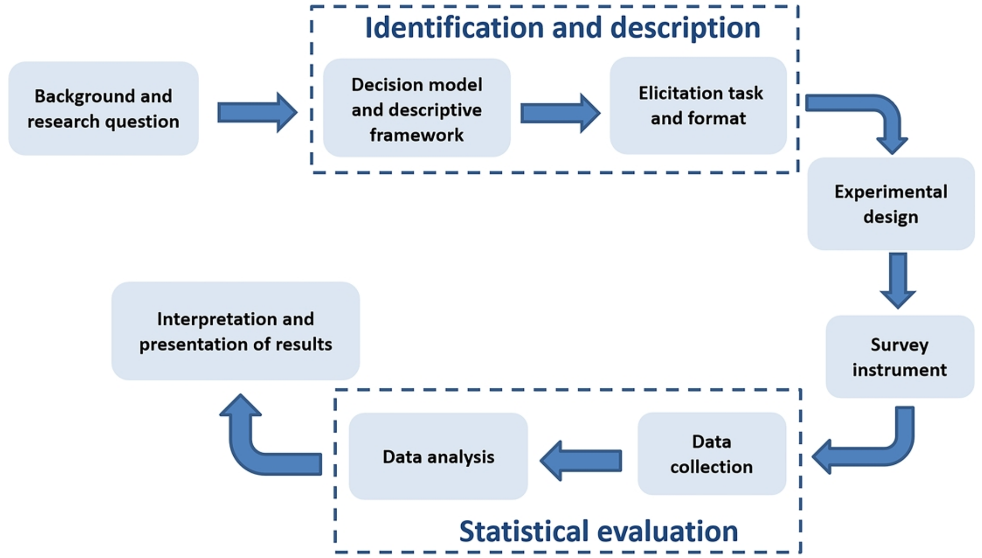

Figure 1: A framework for stated-preference studies

399x234mm (300 x 300 DPI)

Welcome and landing page

What is a successful treatment for a child with a surgical condition?
An online survey of people's preferences

Thank you for your interest in taking part in this survey.

Responses to the survey will help us improve the care of children who have a surgical condition.

If you are completing this survey using a tablet or smartphone, we recommend you use landscape mode.

In total, this survey will take about 15-20 minutes to complete. It will take approximately 10 minutes to work through the instructions and warm up questions, and about 5-10 minutes to complete the survey questions themselves.

Please click next to start.

Nuffield Department of POPULATION HEALTH

FUNDED BY NIHR | National Institute for Health Research

For peer review only - http://bmjopen.bmj.com/site/about/guidelines.xhtml

Screening questions

Screening question 1 of 3:

Please choose the option which *best* describes you:

- ◯ I am a parent or carer of a child who has been treated for a surgical condition
- ◯ I was treated as a child for a surgical condition
- ◯ I am a healthcare professional caring for children with surgical conditions

For peer review only

Screening question 2 of 3:

What is your age (years):

[ ]

Screening question 3 of 3:

What is your gender?

- ◯ Female
- ◯ Male
- ◯ Other
- ◯ Prefer not to say

For peer review only - http://bmjopen.bmj.com/site/about/guidelines.xhtml

Background

Description of the <u>characteristics</u> used in the exercises

We would like you to consider scenarios describing what has happened to an imaginary child after they have been treated for a surgical condition. Before we show you the scenarios, we will take you through the *four characteristics* we will use to describe the scenarios in the exercises. You can read about the characteristics or watch short videos (less than one minute) describing them.

We will ask you some questions about each of these characteristics. **There are no right or wrong answers to these questions,** they are just to help you to get used to the characteristics.

Throughout the survey, the characteristics will be presented in the same order. However, this does not mean that one characteristic is more important than another.

Click next for the first *characteristic.*

Operations (Follow me for a short video)

Most surgical conditions are treated with one or more operations. Some operations that children undergo will be planned at the beginning of that child's treatment, whilst some will be emergencies. How complex an operation is also varies, from minor operations, such as draining an abscess (a pocket of pus under the skin), to more major operations, such as removing sections of intestine (bowel or gut).

In the scenarios you will be shown how many of each of the following types of operation an imaginary child undergoes:

- Planned major operations
- Planned minor operations
- Emergency major operations
- Emergency minor operations

We are only describing **the number of operations** the child had that are **related to their surgical condition**. For example, an operation for appendicitis would not be related.

Please answer the next questions about operations. **There are no right or wrong answers - we are just interested in what you think**.

How many <u>major planned</u> operations do you think are normally needed to treat a child with a <u>broken leg</u>?

No major planned operations	One major planned operation	Two major planned operations	Six major planned operations
○	○	○	○

How many <u>minor planned</u> operations do you think are normally needed to treat a child with a <u>broken leg</u>?

No minor planned operations	One minor planned operation	Two minor planned operations	Six minor planned operations
○	○	○	○

For peer review only - http://bmjopen.bmj.com/site/about/guidelines.xhtml

How many major emergency operations do you think are normally needed to treat a child with a broken leg?

No major emergency operations	One major emergency operation	Two major emergency operations	Six major emergency operations
○	○	○	○

How many minor emergency operations are normally needed to treat a child with a broken leg?

No minor emergency operations	One minor emergency operation	Two minor emergency operations	Six minor emergency operations
○	○	○	○

Infections treated in hospital (Follow me for a short video)

In some cases, an infection such as a collection of pus within the tummy, may occur shortly after an operation, whilst the child is still in hospital. In other cases, an infection may develop whilst the child is at home, but the infection is still related to their surgical condition and is serious enough for them to need treatment in hospital.

For the scenarios in this survey, you will be shown **the number of infections** the imaginary child had **that were related to their surgical condition and needed treatment in hospital**.

Please answer the next question about infections treated in hospital that will help you to get familar with the terminology used in this survey. **Please remember there are no right or wrong answers - we are just interested in what you think**.

How many infections treated in hospital do you think children usually develop after treatment for a broken leg?

No infections treated in hospital	One infection treated in hospital	Two infections treated in hospital	Six infections treated in hospital
○	○	○	○

For peer review only - http://bmjopen.bmj.com/site/about/guidelines.xhtml

Quality of life: The child's quality of life (Follow me for a short video)

In the scenarios we will describe the imaginary child's quality of life as **good, fair, or poor**.

Quality of life is very individual, and what one person thinks of as a good quality of life, someone else may describe as a fair quality of life.

Therefore, we would like you to decide for yourself what you think a good, fair, or poor quality of life for the imaginary child may be.

You might think about whether their condition has had any specific impact on them, for example, resulted in them being incontinent of wee or poo, or need for hospital care or visits. There may also be more broad factors to consider such as how much enjoyment a child gets from day-to-day life, the activities they can do for themselves, and impact on school or home life and family members.

Please read the short story about an imaginary child named Molly and answer the question that follows:

Molly is seven years old and several times a week has accidents where she passes poo in her underwear. Some children at school have started to bully her because of this. As a result, Molly doesn't enjoy school, and often tries to find reasons not to go. Over the last year her development has started to fall behind that of her peers. Molly gets quite stressed and anxious.

Which option below do you think best describes Molly's quality of life?

Good quality of life	Fair quality of life	Poor quality of life
○	○	○

Survival: How long the child survived after their diagnosis (Follow me for a short video)

In each of the scenarios we will describe **how long the imaginary child survived after they were diagnosed** with their surgical condition. This will be presented as one of the following categories:

- Lived more than twenty years, without any expectation that their surgical condition would shorten their life expectancy
- Lived twenty years
- Lived five years
- Lived one year
- Lived six months
- Lived one month

Please answer the next question about survival.

How long do you think children usually survive after being diagnosed with a broken leg?

Lived more than twenty years (normal life expectancy)	Lived twenty years	Lived five years	Lived one year	Lived six months	Lived one month
○	○	○	○	○	○

Practice question paired comparison

Exercise 1

In this exercise, you will be shown a pair of scenarios (**A** and **B**). In each scenario the four *characteristics* describe what has happened to an imaginary child with a surgical condition following their treatment.

We would like you to tell us **whether scenario A or scenario B describes the more successful result of the child's treatment.**

This exercise will be made up of 10 questions each presenting a pair of scenarios. **To make it easier to see the difference between the two scenarios, the characteristics that are the same in both scenarios have a grey background.**

Practice question for Exercise 1

On the next page you will see a practice question.

Which scenario describes the more successful result of the child's treatment?
Click Scenario A or B

Practice task:	Scenario A	Scenario B
Operations	Six major planned operations	No major planned operations
	One minor planned operation	One minor planned operation
	Six major emergency operations	No major emergency operations
	One minor emergency operation	One minor emergency operation
Infections treated in hospital	One infection treated in hospital	One infection treated in hospital
Quality of life	Poor quality of life	Good quality of life
Survival	Lived **one month**	Lived more than twenty years (normal life expectancy)
Click Scenario A or B	Scenario A	Scenario B

Exercise 1 practice question feedback

In the practice question you have just completed, you thought about the characteristics of both scenarios and decided that **Scenario B** was the more successful result of the child's treatment.

Please try to answer all of the following 10 questions by selecting the scenario you think is the **more successful result of the child's treatment.** Try to give each question equal attention.

Remember, there are no right or wrong answers – we are just interested in what you think.

Paired comparisons (participants to complete ten tasks)

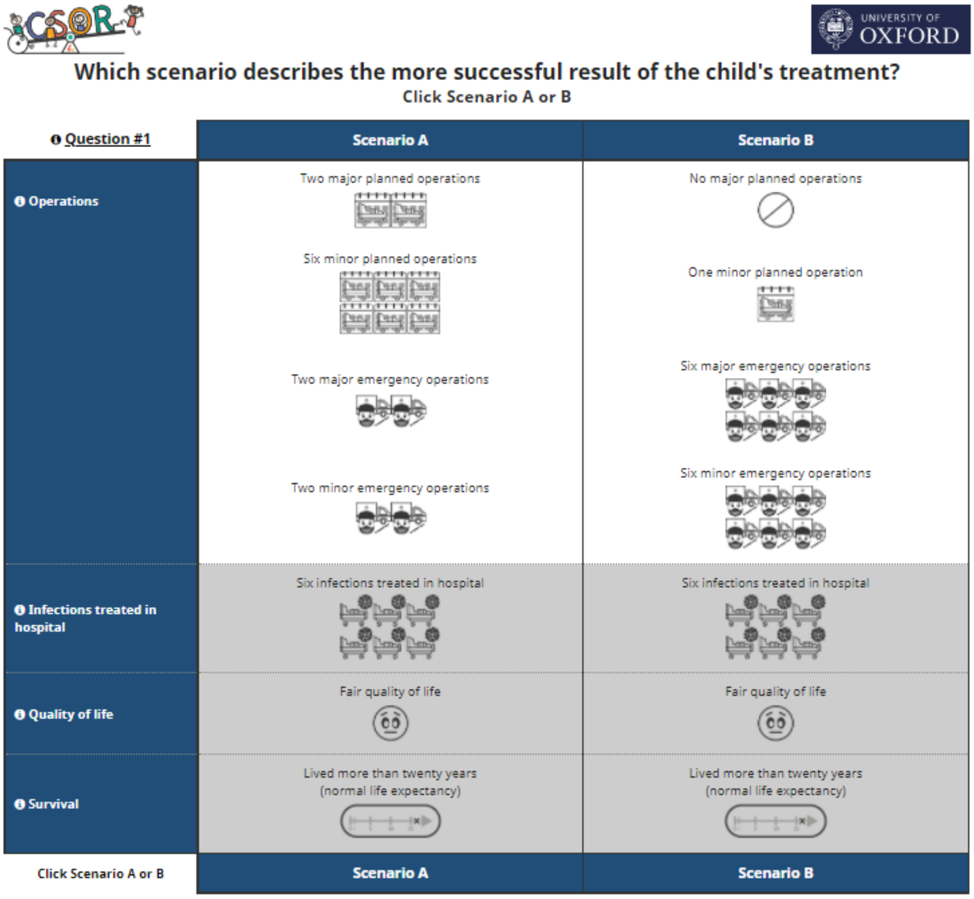

Which scenario describes the more successful result of the child's treatment?
Click Scenario A or B

Question #1	Scenario A	Scenario B
Operations	Two major planned operations<br>Six minor planned operations<br>Two major emergency operations<br>Two minor emergency operations	No major planned operations<br>One minor planned operation<br>Six major emergency operations<br>Six minor emergency operations
Infections treated in hospital	Six infections treated in hospital	Six infections treated in hospital
Quality of life	Fair quality of life	Fair quality of life
Survival	Lived more than twenty years (normal life expectancy)	Lived more than twenty years (normal life expectancy)
Click Scenario A or B	Scenario A	Scenario B

NPEU   Nuffield Department of POPULATION HEALTH   FUNDED BY NIHR National Institute for Health Research

For peer review only - http://bmjopen.bmj.com/site/about/guidelines.xhtml

Practice question kaizen task

Exercise 2

Each question in **Exercise 2** has three parts:

- In the **first** part you will select between two scenarios (**A** and **B**) as you did in Exercise 1.
- In the **second** part you will see one scenario, again described by the four characteristics. Next to some of the characteristics, you will see an alternative option. You will be asked to **choose three characteristics you think will improve the scenario, starting with the one you think will improve it the most**.
- In the **third** part you will select between two scenarios (**A** and **B**) as you did in Exercise 1.

Practice question for Exercise 2

On the next page you will see a practice question.

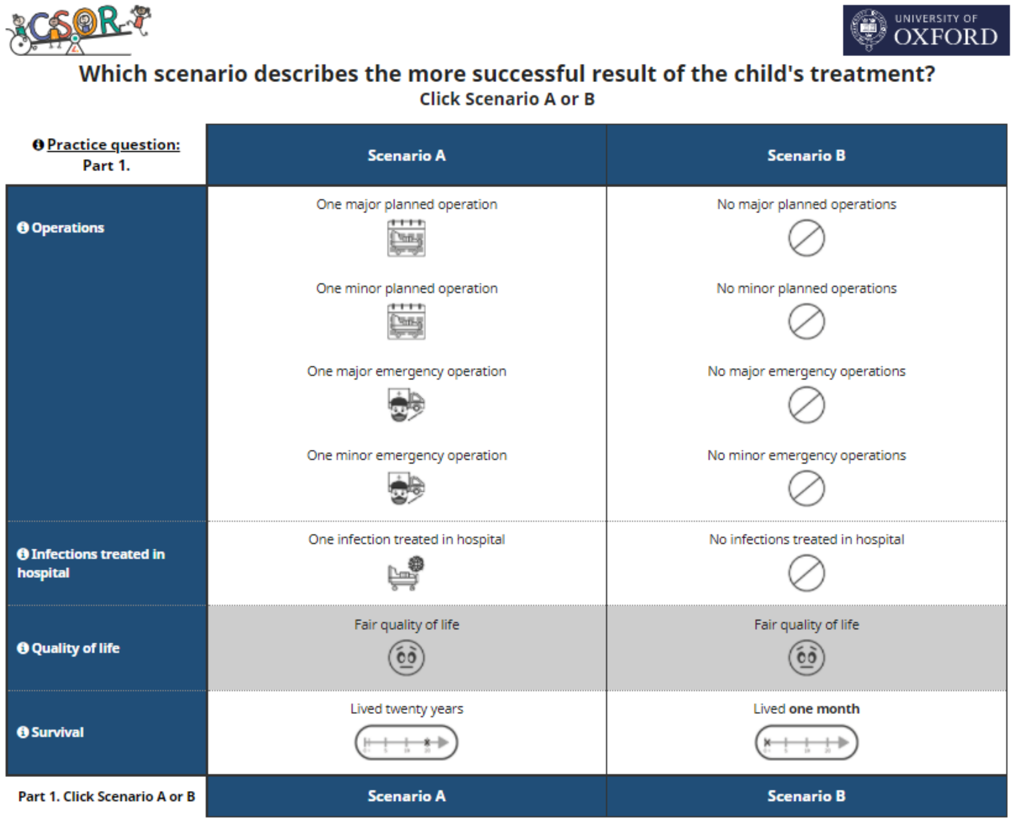

Which scenario describes the more successful result of the child's treatment?
Click Scenario A or B

Practice question: Part 1.	Scenario A	Scenario B
Operations	One major planned operation	No major planned operations
	One minor planned operation	No minor planned operations
	One major emergency operation	No major emergency operations
	One minor emergency operation	No minor emergency operations
Infections treated in hospital	One infection treated in hospital	No infections treated in hospital
Quality of life	Fair quality of life	Fair quality of life
Survival	Lived twenty years	Lived **one month**
Part 1. Click Scenario A or B	Scenario A	Scenario B

Which characteristics would you change to improve scenario A?

(Please choose three characteristics from the second column)

**Practice task:** Part 2.	**Scenario A**	**Select the characteristics in the order you would change them**
**Operations**	One major planned operation	
	One minor planned operation	No minor planned operations
	One major emergency operation	No major emergency operations
	One minor emergency operation	No minor emergency operations
**Infections treated in hospital**	One infection treated in hospital	No infections treated in hospital
**Quality of life**	Fair quality of life	
**Survival**	Lived twenty years	
**Part 2.**	**Scenario A**	**Select the characteristics in the order you would change them**

Clear Selection

iew only

Which scenario describes the more successful result of the child's treatment?
Click Scenario A or B

**Practice Task:** Part 3.	**Scenario A**	**Scenario B**
**Operations**	One major planned operation	No major planned operations
	No minor planned operations	No minor planned operations
	No major emergency operations	No major emergency operations
	No minor emergency operations	No minor emergency operations
**Infections treated in hospital**	No infections treated in hospital	No infections treated in hospital
**Quality of life**	Fair quality of life	Fair quality of life
**Survival**	Lived twenty years	Lived **one month**
**Part 3. Click Scenario A or B**	**Scenario A**	**Scenario B**

evie

Exercise 2 practice question feedback

The practice question you have just completed had three parts:

- In the first part you selected **the Scenario A.**
- In the second part you improved three characteristics of **Scenario A:**
  - **First** from one minor planned operation to no minor planned operations
  - **Second** from one major emergency operation to no major emergency operations
  - **Third** from one minor emergency operation to no minor emergency operations
- In the third part you selected the improved **Scenario A.**

Please try to answer all of the following 3 questions. As in the practice question, in the **first part** of each question you need to select a scenario, in the **second part** you need to make three improvements starting with the one you think is most important, and in the **third part** you need to again select a scenario.

Remember, there are no right or wrong answers – we are just interested in what you think.

Kaizen tasks (participants to complete three tasks)

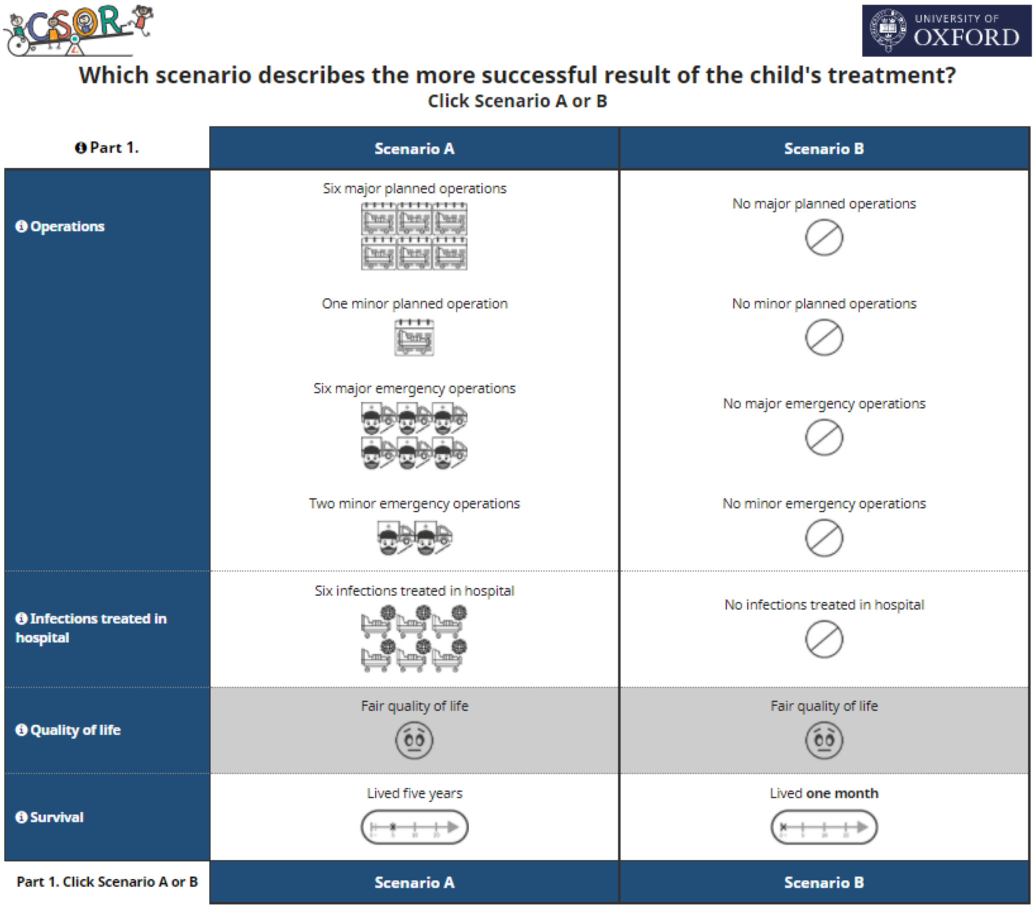

ew only

For peer review only - http://bmjopen.bmj.com/site/about/guidelines.xhtml

Which characteristics would you change to improve scenario A?

(Please choose three characteristics from the second column)

Question 2 Part 2.	Scenario A	Select the characteristics in the order you would change them
Operations	Six major planned operations	
	One minor planned operation	No minor planned operations
	Six major emergency operations	Two major emergency operations
	Two minor emergency operations	
Infections treated in hospital	Six infections treated in hospital	No infections treated in hospital
Quality of life	Fair quality of life	Good quality of life
Survival	Lived five years	
Part 2.	Scenario A	Select the characteristics in the order you would change them

Clear Selection

ew only

For peer review only - http://bmjopen.bmj.com/site/about/guidelines.xhtml

Which scenario describes the more successful result of the child's treatment?

Click Scenario A or B

Part 3.	Scenario A	Scenario B
Operations	Six major planned operations	No major planned operations
	No minor planned operations	No minor planned operations
	Two major emergency operations	No major emergency operations
	Two minor emergency operations	No minor emergency operations
Infections treated in hospital	No infections treated in hospital	No infections treated in hospital
Quality of life	Good quality of life	Fair quality of life
Survival	Lived five years	Lived one month
Part 3. Click Scenario A or B	Scenario A	Scenario B

iew only

For peer review only - http://bmjopen.bmj.com/site/about/guidelines.xhtml

Debriefing questions

Please tell us what you thought about the two different exercises you just answered

	Exercise 1: Choosing between scenario A and B	Leaning towards Exercise 1	About the same	Leaning towards Exercise 2	Exercise 2: Choosing between scenario A and B and select three improvements
Which exercise did you **prefer to complete**?	○	○	○	○	○
Which exercise did you find **easier to understand**?	○	○	○	○	○
Which exercise did you find **easier to complete**?	○	○	○	○	○

Would you like to comment about the two different exercises you just answered?

For peer review only

Demographics questions (parents and carers)

About you

To help us ensure the results are an accurate description of what a successful treatment is for a child with a surgical condition, it is important that we understand who has completed the survey. Please answer the following questions. It will not be possible to identify you from your responses.

Which country do you usually live in?

- ◯ UK
- ◯ Ireland
- ◯ Australia
- ◯ Canada
- ◯ New Zealand
- ◯ United States of America
- ◯ Other: ______

Please select the surgical condition(s) for which your child has been treated:

- ☐ Hirchsprung's disease
- ☐ Necrotising enterocolitis
- ☐ Gastroschisis
- ☐ Oesophageal atresia
- ☐ Congenital Diaphragmatic Hernia
- ☐ Posterior Urethral Valves
- ☐ Other: ______

Please select which age category your child falls into:

- ◯ My child has died
- ◯ Less than 1 year old
- ◯ 1 to 5 years old
- ◯ 6 to 10 years old
- ◯ 11 to 18 years old
- ◯ More than 18 years old

For peer review only - http://bmjopen.bmj.com/site/about/guidelines.xhtml

Please select how you would describe your child's current quality of life:

- Good quality of life
- Fair quality of life
- Poor quality of life
- Prefer not to answer

Are you:

- Single
- Married/Partner
- Separated
- Divorced
- Widowed
- Prefer not to answer

Please indicate which occupational group the main earner in your household belongs to, or which group fits best:

- Managers, directors and senior officials
- Professional/Associate Professional occupations
- Administrative and secretarial occupations
- Skilled trades occupations
- Caring, leisure and other service occupations
- Sales and customer service occupations
- Process, plant and machine operatives
- Elementary occupations
- Pensioner
- Unemployed
- Never employed
- Prefer not to answer

Do you have a Degree or equivalent professional qualification?

- Yes
- No

For peer review only - http://bmjopen.bmj.com/site/about/guidelines.xhtml

Have you experienced serious illness in addition to having a child with a surgical condition?

	Yes	No	Prefer not to answer
Yourself	○	○	○
In your family	○	○	○
As a carer for a family member	○	○	○

For peer review only

For peer review only - http://bmjopen.bmj.com/site/about/guidelines.xhtml

Demographics questions (individuals treated as child for surgical condition)

About you

To help us ensure the results are an accurate description of what a successful treatment is for a child with a surgical condition, it is important that we understand who has completed the survey. Please answer the following questions. It will not be possible to identify you from your responses.

Which country do you usually live in?

- UK
- Ireland
- Australia
- Canada
- New Zealand
- United States of America
- Other:

Please select the surgical condition(s) for which you were treated as a child:

- Hirchsprung's disease
- Necrotising enterocolitis
- Gastroschisis
- Oesophageal atresia
- Congenital Diaphragmatic Hernia
- Posterior Urethral Valves
- Other:

Please indicate which occupational group the main earner in your household belongs to, or which group fits best:

- Managers, directors and senior officials
- Professional/Associate Professional occupations
- Administrative and secretarial occupations
- Skilled trades occupations
- Caring, leisure and other service occupations
- Sales and customer service occupations
- Process, plant and machine operatives
- Elementary occupations
- Pensioner
- Unemployed
- Never employed
- Prefer not to answer

For peer review only - http://bmjopen.bmj.com/site/about/guidelines.xhtml

Do you have a Degree or equivalent professional qualification?

- ◯ Yes
- ◯ No

Have you experienced serious illness in addition to having a child with a surgical condition?

	Yes	No	Prefer not to answer
Yourself	◯	◯	◯
In your family	◯	◯	◯
As a carer for a family member	◯	◯	◯

peer review only

For peer review only - http://bmjopen.bmj.com/site/about/guidelines.xhtml

Demographics questions (healthcare professionals)

About you

To help us ensure the results are an accurate description of what a successful treatment is for a child with a surgical condition, it is important that we understand who has completed the survey. Please answer the following questions. It will not be possible to identify you from your responses.

Which country do you usually live in?

- ◯ UK
- ◯ Ireland
- ◯ Australia
- ◯ Canada
- ◯ New Zealand
- ◯ United States of America
- ◯ Other: ______

What is your role/specialism?

- ◯ Surgeon
- ◯ Anaesthetist
- ◯ Paediatrician
- ◯ Neonatologist
- ◯ Nurse
- ◯ Advanced Nurse Practitioner/Specialist Nurse
- ◯ Other: ______

For surgeon, anaesthetist, paediatrician and neonatologist

Please select your highest current level of training:

- ◯ Consultant or equivalent
- ◯ Registrar or equivalent
- ◯ Foundation or core trainee
- ◯ Prefer not to answer

For nurse, advanced nurse practitioner, specialist nurse

Please select your highest current level of training:

- ◯ Consultant
- ◯ Modern Matron or Chief Nurse
- ◯ Advanced Nurse/Nurse practitioner
- ◯ Nursing specialist or Senior Nurse
- ◯ Newly qualified nurse
- ◯ Prefer not to answer

For peer review only - http://bmjopen.bmj.com/site/about/guidelines.xhtml

Have you experienced serious illness in addition to having a child with a surgical condition?

	Yes	No	Prefer not to answer
Yourself	○	○	○
In your family	○	○	○
As a carer for a family member	○	○	○

For peer review only

For peer review only - http://bmjopen.bmj.com/site/about/guidelines.xhtml

End of survey and thanks

Thank you for taking part in our survey

If you know other people that you think may be interested in taking part, please tell them about our study so they can register here.

Please do not share your link to the survey with others, as this is unique to you.

If you would like receive email updates on this project, as well as other opportunities to support research in child health, please contact the team at csor@npeu.ox.ac.uk. Your information will be kept securely at the University of Oxford and will not be used for any other purpose or shared with others.

If you would like to talk to someone about how completing the survey has affected you, please get in touch with the CSOR team and we will try to put you in contact with one of the many charities that have been supporting this project.

review only

For peer review only - http://bmjopen.bmj.com/site/about/guidelines.xhtml

Supplementary material 2: Experimental design for the pair comparison

		Scenario 1							Scenario 2						
Choice situation	Block	planned_ major	planned_ minor	emergency_ major	emergency_ minor	infection	qol	survival	planned_ major	planned_ minor	emergency_ major	emergency_ minor	infection	qol	survival
1	3	3	4	3	4	4	3	4	4	4	3	1	3	3	5
2	4	1	3	4	2	4	2	1	2	1	1	2	4	3	1
3	4	1	1	1	3	4	1	1	4	1	4	2	3	1	1
4	5	2	1	3	3	4	3	5	4	1	4	3	3	3	1
5	3	4	2	4	3	4	1	4	4	3	4	1	4	3	3
6	5	4	1	2	4	3	3	3	4	1	4	2	4	3	2
7	3	2	1	2	4	4	3	1	2	1	4	3	3	3	5
8	3	1	1	3	3	4	3	1	3	1	4	1	4	1	1
9	1	4	2	3	1	4	3	5	4	3	2	4	4	2	5
10	2	4	1	3	4	1	1	2	4	3	2	2	1	3	2
11	5	2	4	3	2	2	3	3	2	4	3	4	1	2	5
12	1	3	3	1	4	3	1	1	4	1	2	2	3	1	1
13	4	3	3	4	1	2	3	2	3	4	4	4	2	2	1
14	4	2	2	4	2	4	2	3	4	3	2	2	4	2	1
15	3	2	1	4	1	2	2	5	2	1	4	3	1	3	3
16	3	3	4	4	4	2	3	1	4	1	3	4	2	3	2
17	1	1	4	1	1	3	1	1	2	2	2	1	1	1	1
18	3	2	4	2	2	1	3	4	3	1	2	3	1	3	1
19	3	2	3	3	2	1	3	2	2	4	2	1	4	3	2
20	5	2	3	2	3	2	1	1	4	3	2	1	1	1	4
21	2	2	3	4	4	3	3	2	4	1	4	4	2	3	4
22	2	3	2	2	1	3	3	3	3	3	2	3	2	3	1
23	1	4	1	4	3	1	2	3	4	2	3	3	4	1	3
24	4	3	3	3	3	4	3	5	3	4	3	4	1	3	2
25	5	3	1	2	2	4	1	2	3	1	3	1	2	1	4
26	1	2	4	2	4	3	2	2	3	4	2	2	1	2	3

For peer review only

27	1	3	1	2	2	2	2	5	4	1	4	1	2	2	1
28	5	4	1	2	1	1	2	4	4	2	2	1	2	1	1
29	2	3	2	3	2	4	3	4	4	2	4	2	1	2	4
30	5	3	1	3	4	4	2	1	4	2	1	4	2	2	1
31	2	3	1	2	3	3	1	1	3	4	2	1	2	3	1
32	1	3	1	3	1	4	2	3	3	1	3	4	3	3	2
33	5	2	1	4	3	2	3	4	2	3	4	3	4	1	3
34	4	1	2	2	1	3	3	1	4	2	1	1	4	1	1
35	4	1	4	2	2	2	1	1	4	1	1	2	2	2	1
36	4	2	1	2	4	1	3	1	2	1	3	2	3	1	1
37	2	2	1	2	1	3	1	4	2	4	2	3	2	1	2
38	1	2	1	4	4	3	3	4	4	2	4	4	3	2	2
39	3	3	4	2	3	1	2	5	3	4	3	1	2	2	3
40	2	1	1	1	2	2	3	1	4	1	1	1	4	2	1
41	1	1	2	4	4	4	2	1	3	4	3	3	4	2	1
42	5	3	1	4	4	4	3	3	3	2	3	4	1	3	4
43	2	2	2	3	4	2	3	1	3	1	3	1	2	3	4
44	4	2	3	4	3	3	1	1	3	3	4	2	1	3	1
45	2	3	1	4	3	3	2	1	3	2	4	2	3	1	4

For peer review only

Definitions of attribute levels:
planned_major; planned_minor; emergency_major; emergency_minor coded as:
1 = No operations
2 = One operation
3 = Two operations
4 = Six operations

Infection coded as:
1 = No infection treated in hospital
2 = One infection treated in hospital
3 = Two infections treated in hospital
4 = Six infections treated in hospital

Quality of life coded as:
1 = Good quality of life
2 = Fair quality of life
3 = Poor quality of life

Survival coded as:
1 = Lived more than twenty years (normal life expectancy)
2 = Lived twenty years
3 = Lived five years
4 = Lived one year
5 = Lived six months

For peer review only

For peer review only - http://bmjopen.bmj.com/site/about/guidelines.xhtml

BMJ Open

Using stated-preferences methods to develop a summary metric to determine successful treatment of children with a surgical condition: a study protocol

Journal:	*BMJ Open*
Manuscript ID	bmjopen-2022-062833.R1
Article Type:	Protocol
Date Submitted by the Author:	15-May-2022
Complete List of Authors:	Rivero-Arias, Oliver; University of Oxford, Nuffield Department of Population Health<br>Buckell, John; University of Oxford, Health Economics Research Centre. Nuffield Department of Population Health<br>Allin, Benjamin; University of Oxford, National Perinatal Epidemiology Unit. Nuffield Department of Population Health; Chelsea and Westminster Hospital<br>Craig, Benjamin ; University of South Florida<br>Ayman, Goher; University of Oxford, National Perinatal Epidemiology Unit. Nuffield Department of Population Health<br>Knight, Marian; University of Oxford Nuffield Department of Population Health, National Perinatal Epidemiology Unit<br>Collaborative Group, CSOR; University of Oxford, National Perinatal Epidemiology Unit. Nuffield Department of Population Health.
**Primary Subject Heading**:	Surgery
Secondary Subject Heading:	Paediatrics, Health economics, Health policy, Health services research
Keywords:	PAEDIATRIC SURGERY, HEALTH ECONOMICS, HEALTH SERVICES ADMINISTRATION & MANAGEMENT

SCHOLARONE™
Manuscripts

Using stated-preferences methods to develop a summary metric to determine successful treatment of children with a surgical condition: a study protocol

Oliver Rivero-Arias[1], John Buckell[2], Benjamin Allin[1,3], Benjamin M Craig[4], Goher Ayman[1] and Marian Knight[1] on behalf of the CSOR Collaborative Group

1. National Perinatal Epidemiology Unit, Nuffield Department of Population Health, University of Oxford, Oxford, UK
2. Health Economics Research Centre, Nuffield Department of Population Health, University of Oxford, Oxford, UK
3. Chelsea and Westminster Hospital, London, UK
4. Department of Economics, University of South Florida, Tampa, USA

Corresponding author:
Associate Professor Oliver Rivero-Arias
National Perinatal Epidemiology Unit
Nuffield Department of Population Health
Old Road Campus
University of Oxford
OX3 7LF
Email: oliver.rivero@npeu.ox.ac.uk

For peer review only

For peer review only - http://bmjopen.bmj.com/site/about/guidelines.xhtml

Abstract

Introduction: Wide variation in the management of key paediatric surgical conditions in the UK has likely resulted in outcomes for some children being worse than they could be. Consequently, it is important to reduce unwarranted variation. However, major barriers to this are the inability to detect differences between observed and expected hospital outcomes based upon the case-mix of the children they have treated, and the inability to detect variation in significant outcomes between hospitals. A stated-preference study has been designed to estimate the value key stakeholders place on different elements of the outcomes for a child with a surgical condition. This study proposes to develop a summary metric to determine what represents successful treatment of children with surgical conditions.

Methods and analysis: Preferences from parents, individuals treated for surgical conditions as infants/children, health care professionals and members of the public will be elicited using paired comparisons and kaizen tasks. A descriptive framework consisting of seven attributes representing types of operations, infections treated in hospital, quality of life and survival was identified. An experimental design has been completed using a D-efficient design with overlap in three attributes and excluding implausible combinations. All participants will be presented with an additional choice task including a palliative scenario that will be used as an anchor. The survey will be administered online. Primary analysis will estimate a mixed multinomial logit model. A traffic light system to determine what combination of attributes and levels represent successful treatment will be created.

Ethics and dissemination: Ethics approval to conduct this study has been obtained from the Medical Sciences Inter-Divisional Research Ethics Committee (IDREC) at the University of Oxford (R59631/RE001-05). We will disseminate all of our results in peer-review publications and scientific presentations. Findings will be additionally disseminated through relevant charities and support groups and professional organisations.

For peer review only - http://bmjopen.bmj.com/site/about/guidelines.xhtml

Strengths and limitations of this study

- Our descriptive system has been developed using formative research best practice guidance.
- Our experimental design employs attribute level overlap and exclude implausible combinations of attribute and levels in the context of children with a surgical condition.
- All participants will complete a choice task paired comparison with one of the alternatives representing a palliative scenario that will be used as an anchor.
- Preference data from different stakeholders relevant to the decision context will be available to estimate the final summary metric.
- Given that children with surgical conditions are relatively few in number, data collection may present challenges, in particular for the identification of parents and health care professionals, which will be mitigated using a thorough recruitment strategy.

For peer review only

1 Introduction

In England and Scotland there are currently 24 Trusts/Health Boards commissioned to provide specialised surgery in children [1]. Specialised surgery in children includes:

a) Management of rare surgical conditions in children
b) Provision of specified specialised surgical procedures during childhood
c) Surgery in neonates
d) Surgical management or procedures for more common paediatric surgical conditions when a child requires specialist pre-operative, anaesthetic or post-operative care (simple surgical procedures in children with complex medical needs)

Many of the conditions falling under the remit of *specialised surgery in children* commissioning affect only a few hundred children in England and Scotland each year, and what little is known about these children's long-term health and wellbeing suggests that even after treatment, they have significant ongoing healthcare needs [2-8]. Widespread variation in management of children with these conditions currently occurs [2-7], but due to the rarity of the conditions, it has not to date been possible to identify how much of this variation is unwarranted or associated with variation in outcome. To identify unwarranted variation in management and outcome between centres providing *specialised surgery in children* it is necessary to develop mechanisms that will:

1. Collect accurate, unbiased data about children treated in individual centres
2. Combine data from children with different conditions in a way that enables meaningful outcomes analysis
3. Enable adjustment for case-mix factors affecting centres' outcomes

The NIHR funded study "Improving unwarranted variation in outcomes of children's surgery through a new Children's Surgery Outcome Reporting system using routinely available data (CSOR)" investigates whether one unified system is capable of addressing these three issues and therefore reducing unwarranted variation in surgical care. This protocol paper describes the study design of one of the CSOR sub-studies, tackling the second barrier.

For peer review only - http://bmjopen.bmj.com/site/about/guidelines.xhtml

The gold standard approach for comparing outcomes of interest to patients with a specific condition is to use a core outcome set (COS) [9]. Several COS have recently been developed that are relevant to children with a surgical condition [10-13]. Development of these COSs has highlighted that whilst there are attributes of successful treatment that are unique to each individual condition, there are also attributes that are common across conditions. A single outcome measure that combines these common attributes with attributes that accurately reflected the condition specific elements of each COS (e.g. a measure of quality of life) would be able to provide a meaningful assessment of how successful the treatment of a child with any surgical condition has been. The aim of this study is therefore to develop an algorithm to assist in combining these attributes into one summary metric, and therefore determining what constitutes successful treatment of children with a surgical condition. This metric would facilitate combination of data from children with different conditions enabling meaningful outcomes analysis.

Whether a certain combination of common core outcomes across conditions indicates a successful or unsuccessful treatment depends on the value that relevant stakeholders place on the different elements of the core outcomes. Economists employ preference elicitation techniques to determine such values [14]. Stated preference techniques such as discrete choice experiments (DCE) are well-suited to understand the value of potential combinations of core outcomes of paediatric surgery [15]. A DCE is an experiment with choice tasks that elicit preferences indicating how individuals value attributes of alternatives in a decision scenario [15]. The value of a scenario depends on the levels associated to attributes, which are the characteristics of health, treatments or health care services being evaluated [15]. During a DCE, participants are presented with a number of scenarios and are asked to choose their preferred option, trading off among the attributes. DCEs can take different formats, but paired comparisons, where the participant is presented with two or more scenarios and asked to choose one, are most widely used [16]. Other alternatives to paired comparisons exist including best-worst scaling and more recently kaizen tasks [17, 18]. Participants' choices in stated preferences exercises are analysed using discrete choice models, where choices are associated with combinations of attributes and levels to understand participants' preferences (i.e., their relative importance).

For peer review only - http://bmjopen.bmj.com/site/about/guidelines.xhtml

This is a protocol for a stated-preference study designed to estimate the value key stakeholders place on different combinations of health and care outcomes following treatment of a child with a surgical condition. Stakeholders will be presented with a series of paired comparisons and novel kaizen tasks to elicit their preferences. The final product of the collected data will be an algorithm to determine whether the treatment of a child with a surgical condition has been "successful" or "unsuccessful". The use of stakeholder preferences to help in health care decision-making by policymakers has increased considerably across most developed jurisdictions [16]. This has been accompanied by best practice guidance for developing such studies, applied herein [19-22].

1.1 Aims

To understand how parents, guardians and health care professionals caring for children with a surgical condition, individuals treated for surgical conditions as children, and members of the general public value common health and care outcomes following treatment of a child with a surgical condition. Specifically:

A. To estimate the relative importance of key health outcomes following treatment for a surgical condition in childhood for multiple conditions using a paired comparison and a novel kaizen task.
B. To compare preferences between the two sources of preference data and type of participant in the context of children with a surgical condition.
C. To estimate an algorithm using weights derived from the relative importance estimates in A to derive a summary metric that categorises outcomes following surgery in childhood into "successful" or "unsuccessful" outcome.

2 Methods and analysis

2.1 Overview of the framework for the stated-preference study

Figure 1 describes the framework and different phases that will be followed to conduct this study. This protocol describes the following sections: 1) identification and description, 2) experimental design, 3) survey instrument, and 4) statistical analysis.

For peer review only - http://bmjopen.bmj.com/site/about/guidelines.xhtml

2.2 Identification and description

2.2.1 Decision model and descriptive framework

We have followed the most recent guidance on formative research for the identification of attributes and levels for the descriptive system used in this study [22]. Our decision problem explores how to best conceptualise what constitutes a more successful outcome following treatment for a surgical condition in childhood from the values that relevant stakeholders place on key core outcomes across paediatric surgical conditions. Our decision model hypothesises that a successful treatment for a child with a surgical condition can be represented by a combination of characteristics or attributes. In this study, we define attributes as core health outcomes included in available COSs relevant to paediatric surgery. The attributes and associated levels that describe potential outcomes following treatment define our descriptive framework. We used literature reviews, interviews with parents and paediatric surgeons, and group discussions with our Parent Advisory Group (PAG) to determine the descriptive system for the final survey instrument. Our PAG consists of over 100 parents and family of children who have undergone early surgery for conditions including Hirschsprung's disease, gastroschisis, exomphalos, short bowel and necrotising enterocolitis.

An initial list of conceptual attributes were identified through a review of published COS relevant to paediatric surgery. Relevant COS have been developed for children with Hirschsprung's disease, gastroschisis and appendicitis, as well as for children receiving neonatal care in a high-income setting [10-13]. Each of these COS were developed using a combination of literature reviews, an online Delphi process, and consensus meetings, and included in their stakeholder groups, clinicians, allied health professionals, parents, and children or adults previously treated for the target condition. We also conducted focussed discussions with our PAG who had the opportunity to comment on the COS identified in the literature review. The core outcomes identified in the above mentioned conditions are presented in Table 1.

For peer review only - http://bmjopen.bmj.com/site/about/guidelines.xhtml

Table 1: Summary of identified core outcomes in neonatal conditions

Category	Core outcome	Included in core outcome set?			
		Hirschsprung's Disease	Gastroschisis	Neonatal care	Appendicitis
Survival	Survival	x	x	x	x
Quality of life	Quality of life	x	x	x	x
	Psychological stress	x			x
	Time away from full activity				x
Adverse events	Unplanned reoperation	x			x
	Number of operations		x		
	Severe gastrointestinal complication		x		
	Retinopathy of prematurity			x	
	Chronic lung disease			x	
	Bowel obstruction				x
	Readmission				x
	Length of hospital stay				x
	Significant infection		x	x	
	Hirschsprung's Associated Enterocolitis	x			
	Necrotising enterocolitis			x	
	Wound infection				x
	Wound complication				x
	Intra-abdominal abscess				x
Condition specific	Faecal incontinence	x			
	Bowel function score	x			
	Voluntary bowel movements	x			
	Urinary incontinence	x			
	Permanent stoma	x			
	Growth		x		
	Time on parenteral nutrition		x		
	Liver disease		x		
	Brain injury on imaging			x	
	Motor/cognitive/visual/hearing ability			x	
	Antibiotic failure				x
	Negative appendicectomy				x
	Recurrent appendicitis				x

For peer review only

For peer review only - http://bmjopen.bmj.com/site/about/guidelines.xhtml

An iterative process to the identification of attributes was followed. In a first step, conducted by the clinical team (BA, MK), overlap in outcomes of importance was identified between the four COS. Each COS also identified outcomes that were relevant only to the condition of interest, and not represented in the other COSs. In a second step, we reviewed these condition-specific outcomes with our PAG in order to determine how best to represent them in the descriptive system. The group concluded that these condition-specific outcomes were highly likely to impact the child's overall quality of life and would therefore be adequately represented through an overarching attribute of quality of life. Three outcome categories including survival, adverse events, and quality of life were common to all four relevant COSs and therefore selected as the initial set of attributes for the descriptive system. Similar outcomes have also been identified as important in other developed paediatric COS [23, 24]. The specific adverse events identified from each COS were also discussed with our PAG and the CSOR paediatric surgeons in a group discussion. The PAG and surgeons agreed that the main adverse events were better summarised as operations, and hospitalisations due to significant infections. Therefore, four core attributes were identified at the end of the second step. A description of each attribute is given next:

1. **Operations**

Most surgical conditions are treated with one or more operations. Some operations that a child might undergo will be planned at the beginning of the child's treatment, whilst some will be emergency, or unplanned operations. The complexity of each operation also varies, from minor operations, such as draining an abscess, to more major operations, such as removing sections of intestine (bowel or gut). For the purpose of the preference study, the number of operations will be presented within four different attributes according to type of operation.

2. **Whether the child has an infection treated in hospital after their operation**

Each of the COSs identified as relevant to paediatric surgery include condition-specific significant infective complications, such as enterocolitis and intra-abdominal abscess. Some also include a more generalised measure of significant infection, *sepsis.* For the purposes of the preference study, the infective complications included in each COS will be represented by the attribute infections treated in hospital, and the levels will define the frequency of infections.

3. **The child's quality of life**

For peer review only - http://bmjopen.bmj.com/site/about/guidelines.xhtml

Each of the COSs included the outcome quality of life, whilst some also specifically included outcomes relating to psychological wellbeing. There are multiple instruments to measure quality of life in children, but they have not been validated in children with a surgical condition. These tools generally describe multiple domains, including social functioning, physical functioning, and psychological wellbeing, with their output generally reported in a continuous manner. However, for the purposes of our descriptive system, quality of life will be categorised as good, fair or poor. The impact of key condition-specific outcomes will be reflected in the child's overall quality of life.

4. How long the child survives after their diagnosis

Although death is relatively uncommon following most surgery in childhood, it is such a significant outcome that all four COSs relating directly to childhood surgery, and the majority of paediatric COSs include it. For the purposes of the descriptive system, this outcome will be presented positively as survival.

The identification of the attribute levels also employed an iterative process. Firstly, we reviewed the epidemiological data available for each of the attributes to guide the range that could be presented to participants. Existing large-scale cohort studies describing the outcomes for children with a range of conditions treated under the specialised surgery in children commissioning, and affecting different regions of the body, modes of presentation, and urgencies of intervention were reviewed [25-38]. A researcher (BA) extracted point estimates and associated measures of uncertainty for each of the attributes. In discussions with two other researchers (OR-A, BC) initial deterministic ordinal levels for each attribute were developed. The selection of levels considered the potential participant cognitive burden and the ability to test appropriate functional forms hypothesis (e.g., linear, quadratic) for quantitative attributes. This initial list of ordinal levels was discussed with paediatric surgeons in a group meeting to ensure their clinical appropriateness and suggest changes to the wording. The language used to describe the levels was refined further following review by members of the PAG. Finally, we conducted three think aloud exercises with different parents who completed a mock choice task of the survey instrument with two of the researchers (OR-A, JB). Parents were given the opportunity during these interviews to comment on the wording used for the attributes and associated levels. The proposed attributes and attribute levels are described in the descriptive framework in Table 2.

For peer review only - http://bmjopen.bmj.com/site/about/guidelines.xhtml

Table 2: Study descriptive framework

**Attributes**	**Attribute Levels**
P*lanned major* operations related to the condition	- No planned major operations<br>- One planned major operation<br>- Two planned major operations<br>- Six planned major operations
P*lanned minor* operations related to the condition	- No planned minor operations<br>- One planned minor operation<br>- Two planned minor operations<br>- Six planned minor operations
Emergency major operations related to the condition	- No emergency major operations<br>- One emergency major operation<br>- Two emergency major operations<br>- Six emergency major operations
Emergency minor operations related to the condition	- No emergency minor operations<br>- One emergency minor operation<br>- Two emergency minor operations<br>- Six emergency minor operations
Infections treated in hospital	- No infections treated in hospital<br>- One infection treated in hospital<br>- Two infections treated in hospital<br>- Six infections treated in hospital
Child's quality of life	- Good quality of life<br>- Fair quality of life<br>- Poor quality of life
How long the child survived after their diagnosis	- More than twenty years, without any expectation that their surgical condition would shorten their life expectancy<br>- Twenty years<br>- Five years<br>- One year<br>- Six months<br>- One month

2.2.2 Elicitation task and format

Two elicitation formats will be used in this study to estimate preferences: a paired comparison and a kaizen task. Each paired comparison will include two scenarios describing combinations of treatment outcomes of a child with a surgical condition without an opt-out option. An example is presented in Supplementary material 1. This type of choice task is the most widely used format in health preference research [16] and has been used previously to elicit preferences for outcomes of surgery [39-41].

Recent work has reported that valuation can be problematic in the context of child health [42, 43]. Eliciting values to inform decision making at the start of life or early childhood requires stakeholders to

For peer review only - http://bmjopen.bmj.com/site/about/guidelines.xhtml

complete tasks from someone else's point of view. There is some evidence that when the tasks in an elicitation exercise refer to someone else's instead of their own preferences, individuals find the exercise strenuous. This is accentuated when the perspective is that of a newborn babies or a young individual [42]. In a paired comparison, it is easy to understand that choosing between two undesirable outcomes of paediatric surgery could be distressing to stakeholders. Moreover, members of the general public may find it both distressing and unfamiliar as they find it difficult to relate to the decision context [44]. This in turn can affect the preferences elicited in paired comparisons.

In addition to the paired comparisons in this study, we will therefore also administer a series of kaizen tasks to participants (supplementary information 1). Each kaizen task begins with a paired comparison between a single profile and a palliative one (i.e. no operations and no infections, but the child has fair health and dies within one month). Next, the respondent makes three improvements to the single profiles. After these improvements, the respondent completes a final paired comparison between the improved profile and a palliative one. Respondents may find the task of improving a child's health to be more engaging than choosing between two diverse outcomes.

2.3 Experimental design

A preliminary experimental design has been completed for both elicitation exercises using a three-step approach: 1) generation of a design for the paired comparison task and 2) selection of pairs for the first and third part of the kaizen task and 3) selection of profiles for the second part of the kaizen task.

Our preliminary design has employed a *D-efficient* design to identify the combination of pairs to present in the paired comparison [15]. This is a procedure for generating choice tasks (for respondents) in a way that maximises the statistical efficiency of the choice models that will be estimated. Several restrictions were imposed including "overlaps" in three attributes to reduce cognitive burden (similar attribute levels between pairs) and implausible combinations of outcomes. These combinations were identified by the CSOR surgical team and included:

a. Reject scenarios in which quality of life is good, the child survived for less than one year, and the child underwent 20 or more operations

For peer review only - http://bmjopen.bmj.com/site/about/guidelines.xhtml

b. Reject scenarios in which the child underwent any number of minor operations, but no major operations, and survived for less than 20 years

c. Reject scenarios in which the child underwent fewer than six minor operations, no major operations, and quality of life is low

d. Reject scenarios in which quality of life is good, the child survived for less than six months, and the child underwent any major operations

A candidate-set was created including these restrictions and used as the initial candidates in Ngene [45]. We generated a preliminary design with 45 choice tasks divided into five blocks to which participants will be randomly allocated i.e. nine choice tasks in each block. Participants will be randomised to one of these five blocks and the order of the pair in each choice task will also be randomised.

An additional choice task was added to all blocks, which serves as an "anchor". This anchor was a palliative profile defined as having no operations, no infections, fair quality of life and a survival of one month. This anchor will be used to facilitate comparisons between paired comparison and kaizen responses and also when developing the final algorithm for CSOR. The experimental design is presented in supplementary material 2.

The experimental design for the kaizen task was constructed directly from the pairs in supplementary material 2. Given each pair, an initial profile of the kaizen task was constructed from the worst attributes found in the pair. Likewise, the four possible improvements were defined to be the best attributes found in the pair. Therefore, the preference path captured by the kaizen task should agree with the paired comparison response (i.e. the path passes through profile chosen in the paired comparison before passing through the profile not chosen in the paired comparison).

2.4 Survey instrument

The survey will be administered online and will be programmed in Oxford University servers with an open source platform. The survey will consist of an initial participant information and consent form, followed by a general welcome, three screening questions, an introduction to the research question

For peer review only - http://bmjopen.bmj.com/site/about/guidelines.xhtml

and description of attributes (provided in both written and short video formats). For each attribute, respondents will answer warm-up tasks to give their view on the attribute for a hypothetical condition (see supplementary material 1). This will be followed by the preference elicitations starting with the 10 paired comparisons and then the three kaizen tasks. For both tasks, participants will have the opportunity to complete a practice question. At the end of the elicitation tasks, participants will be asked three debriefing questions covering which exercise they found easier to complete, prefer to complete and easier to understand. Finally, a set of demographic questions will be collected including experience with neonatal/childhood surgical conditions, employment status and education qualifications. For health care professionals we will also ask their job title and level of professional experience with neonatal/childhood surgical conditions.

A preliminary mock survey has been completed and is presented in supplementary material 1. In developing the instrument, preliminary testing was undertaken to maximise user understanding as described in section 2.2.1.

2.5 Statistical evaluation

2.5.1 Data collection, recruitment strategy and sampling

The survey instrument will be completed by 1) a sample of parents of children with a surgical condition, 2) participants who had treatment for a surgical condition as a baby or a child, 3) health professionals caring for those who undergo surgery in childhood and 4) members of the general public. Main data collection will commence in October 2021.

Recruitment materials will be distributed via existing contacts e.g. by our PAG, by the project's 'experts by experience', and by the project's healthcare professional team members; registers and mailing lists of support groups, charities and professional groups/bodies; and open advertising through support groups, charities and professional groups/bodies' communication channels e.g. Twitter, Facebook, e-newsletters, and websites. Distribution to health professionals will be via professional bodies. We will also include information on our project website and Twitter account, and advertise via Facebook to share the opportunity as widely as possible. These recruitment strategies have been successfully used in previous quantitative and qualitative studies [8, 10, 11, 46, 47].

For peer review only - http://bmjopen.bmj.com/site/about/guidelines.xhtml
For peer review only

We will use an online panel company to invite adult members of the general public to complete the survey. We will use quotas and a target recruitment strategy to ensure the sample is representative of the UK general population in terms of gender, age, social grade and nation.

In this study, we aim to collect 200 responses from parents and individuals with a surgical condition and 200 responses from health care professionals (total of 400) over a three-month period. We are also aiming to collect 400 responses from members of the general public. This is based on typical sample sizes in the health literature and on simple minimum sample size principles [48]. Since there are no prior studies, it is not possible to obtain prior values for accurate power calculations. However, we will conduct a pilot study of around 80 individuals to assess whether our choice models will have sufficient power to detect significant differences.

The pilot study will also be used to assess the feasibility of the survey instrument in terms of finding programming errors and the process of data capture. Only participants recruited from the UK will be included in the final analysis.

2.5.2 Data analysis

In the primary analyses, we will estimate a mixed multinomial logit model with individual-clustered standard errors. Unlike the conditional logit, the mixed specification relaxes the assumption of preference homogeneity by allowing individual-specific random parameters [49]. In the secondary analyses, we will explore preference heterogeneity further by estimating heteroscedastic and latent-class logit models. Although the secondary analysis of preference heterogeneity is more descriptive, we hypothesise that some respondents follow lexicographic processes and that respondents who complete the survey or task more quickly may exhibit greater variance in their responses than respondents who take more time to complete the survey or task. Other behaviours (e.g., task sequence, object position) may also be related to stochastic processes.

In the primary analysis, we will test the following hypotheses:

1. Coefficients are logically consistent and with expected directions.
2. The coefficient for the anchor scenario is negative.
3. Survival and quality of life matter significantly more than the remaining attributes

For peer review only - http://bmjopen.bmj.com/site/about/guidelines.xhtml

4. Survival matters more if child lives in good quality of life and survival matters less if child lives in poor quality of life.

5. Reductions in major planned or emergency operations matter more than reductions in minor or emergency operations.

6. We will observe differences among preferences from the different types of participants but not necessarily between elicitation tasks.

Each type of participant (parents, people who were treated for a surgical condition, health care professionals and members of the general public) will have completed the paired comparison and kaizen tasks. Therefore, eight sets of preference data will be available for analysis. We will compare preferences between types of respondent within each elicitation tasks using two approaches. First, latent scale coefficients of the attribute levels will be rescaled using the coefficient for the anchor scenario and predicted choice probabilities for types of respondent compared using mean square and absolute errors. Second, we will examine the relative attribute importance (RAI) scores by attribute. This involves estimating the range of level combinations for each attribute and applying an attribute-based normalisation to enable comparisons [50]. A similar approach will be used to compare preference evidence between elicitation tasks.

The decision about which coefficients to use in the final algorithm for CSOR will be made by the CSOR Co-investigator Group based on the results of the primary and secondary analyses, feedback from participants, and the face validity of the coefficients obtained.

For the final selected model with rescaled coefficients, we will predict the distribution of utilities of all possible combinations of attribute and levels. This distribution will be used to determine the likelihood of a combination to be considered successful or unsuccessful using a traffic light system: green area (high chance to be successful), amber area (uncertainty about success) and red area (not successful). The external validity of this algorithm will be evaluated in a separate study.

This study is expected to be completed by December 2022.

For peer review only - http://bmjopen.bmj.com/site/about/guidelines.xhtml

2.6 Ethics and dissemination

2.6.1 Ethical considerations

Ethics approval to conduct this study has been obtained from the Medical Sciences Inter-Divisional Research Ethics Committee (IDREC) at the University of Oxford (R59631/RE001-05). Informed consent will be obtained for all participants at the start of the survey.

2.6.2 Dissemination

We will disseminate all of our results in peer-review publications and scientific presentations. A lay summary of the findings will be created using our PAG and circulated to parent support networks and the British Association of Paediatric Surgeons, via social media and on the project website.

2.7 Patient and Public Involvement

Please see section 2.2.

For peer review only

For peer review only - http://bmjopen.bmj.com/site/about/guidelines.xhtml

Footnotes

Author's contributions: OR-A and MK developed the original idea for the study; OR-A, JB, BC, BA and MK were responsible for the study design. OR-A, JB, BA and GA conducted the formative research component informing the development of attributes and levels for the descriptive system. JB, BC and OR-A carried out the experimental design. OR-A and BC implemented the online survey instrument. OR-A drafted the manuscript; all authors revised and approved the final manuscript. All members of the CSOR Collaborative Group participated in the overall programme design and the development and testing of the survey instrument.

Funding statement: This study is funded by the National Institute of Health Research (NIHR) Health Services and Delivery Research (HS&DR) Programme (project reference NIHR 127844). MK is an NIHR Senior Investigator.

Competing interest statement: None

Word count: 4,022

Acknowledgments: We are grateful to our Parental Advisory Group and the paediatric surgeons for their valuable contributions and help with the descriptive system. We would like to thank the participants of our think-aloud exercise, whose suggestions refined the final descriptive system and improved the online survey instrument. We are indebted to Lisa Hinton and Karolina Kuberska for their thoughtful comments on earlier versions of the survey instrument.

CSOR Collaborative Group

Alexander Macdonald (Chelsea & Westminster Hospital), Anna May Long (Cambridge University Hospitals NHS Foundation Trust), Gregor Walker (Royal Hospital for Children, Glasgow), Kokila Lakhoo (Oxford University Hospitals NHS Foundation Trust, University of Oxford), Nigel Hall (Southampton General Hospital and University of Southampton), Nick Lansdale (Royal Manchester Children's Hospital), Simon Kenny (Alder Hey Children's Hospital NHS Foundation Trust), Stefano Giuliani (Great Ormond Street Hospital), Timothy Bradnock (Royal Hospital for Children, Glasgow),

For peer review only - http://bmjopen.bmj.com/site/about/guidelines.xhtml

For peer review only

Joanne Shepherd (Patient Representative), Karolina Kuberska (The Healthcare Improvement Studies Institute (THIS Institute), University of Cambridge), Lisa Hinton (The Healthcare Improvement Studies Institute (THIS Institute), University of Cambridge), University of Cambridge), Goher Ayman (National Perinatal Epidemiology Unit, University of Oxford), Benjamin Allin (National Perinatal Epidemiology Unit, University of Oxford & Chelsea and Westminster Hospital), Charles Opondo (National Perinatal Epidemiology Unit, University of Oxford), Crispin Jenkinson (Health Services Research Unit, University of Oxford), John Buckell (Health Economics Research Centre, University of Oxford), Marian Knight (National Perinatal Epidemiology Unit, University of Oxford), Rema Ramakrishnan (National Perinatal Epidemiology Unit, University of Oxford), Oliver Rivero-Arias (National Perinatal Epidemiology Unit, University of Oxford)

For peer review only

For peer review only - http://bmjopen.bmj.com/site/about/guidelines.xhtml

3 References

1. NHS England. *E02. Specialised Surgery in Children*. 2022 15 May 2022]; Available from: https://www.england.nhs.uk/commissioning/spec-services/npc-crg/group-e/e02/.
2. Bradnock, T.J., et al., *Hirschsprung's disease in the UK and Ireland: incidence and anomalies.* Arch Dis Child, 2017. **102**(8): p. 722-727.
3. Owen, A., et al., *Gastroschisis: a national cohort study to describe contemporary surgical strategies and outcomes.* J Pediatr Surg, 2010. **45**(9): p. 1808-16.
4. Allin, B., et al., *A UK wide cohort study describing management and outcomes for infants with surgical Necrotising Enterocolitis.* Sci Rep, 2017. **7**: p. 41149.
5. Brownlee, E., et al., *Current epidemiology and antenatal presentation of posterior urethral valves: Outcome of BAPS CASS National Audit.* J Pediatr Surg, 2019. **54**(2): p. 318-321.
6. Long, A.M., et al., *Early population-based outcomes of infants born with congenital diaphragmatic hernia.* Arch Dis Child Fetal Neonatal Ed, 2018. **103**(6): p. F517-f522.
7. Burge, D.M., et al., *Contemporary management and outcomes for infants born with oesophageal atresia.* Br J Surg, 2013. **100**(4): p. 515-21.
8. Allin, B.S.R., et al., *Outcomes at five to eight years of age for children with Hirschsprung's disease.* Arch Dis Child, 2020.
9. Williamson, P.R., et al., *The COMET Handbook: version 1.0.* Trials, 2017. **18**(Suppl 3): p. 280.
10. Sherratt, F.C., et al., *Core outcome set for uncomplicated acute appendicitis in children and young people.* Br J Surg, 2020. **107**(8): p. 1013-1022.
11. Allin, B.S.R., et al., *NETS(1HD) study: development of a Hirschsprung's disease core outcome set.* Arch Dis Child, 2017. **102**(12): p. 1143-1151.
12. Allin, B.S.R., et al., *Development of a gastroschisis core outcome set.* Arch Dis Child Fetal Neonatal Ed, 2019. **104**(1): p. F76-F82.
13. Webbe, J.W.H., et al., *Core outcomes in neonatology: development of a core outcome set for neonatal research.* Arch Dis Child Fetal Neonatal Ed, 2020. **105**(4): p. 425-431.
14. Weernink, M.G.M., et al., *A Systematic Review to Identify the Use of Preference Elicitation Methods in Healthcare Decision Making.* Pharmaceutical Medicine, 2014. **28**(4): p. 175-185.
15. Hensher, D.A., J.M. Rose, and W.H. Greene, *Applied Choice Analysis*. 2nd edition ed. 2015, Cambridge: Cambridge University Press.
16. Soekhai, V., et al., *Discrete Choice Experiments in Health Economics: Past, Present and Future.* Pharmacoeconomics, 2019. **37**(2): p. 201-226.
17. Louviere, J.J., T.N. Flynn, and A.A.J. Marley, *Best-worst scaling: methods and applications*. 2015, Cambridge: Cambridge University Press.
18. Craig, B.M., K. Rand, and J.D. Hartman, *Preference Paths and Their Kaizen Tasks for Small Samples.* Patient, 2021.
19. Bridges, J.F., et al., *Conjoint analysis applications in health--a checklist: a report of the ISPOR Good Research Practices for Conjoint Analysis Task Force.* Value Health, 2011. **14**(4): p. 403-13.
20. Hauber, A.B., et al., *Statistical Methods for the Analysis of Discrete Choice Experiments: A Report of the ISPOR Conjoint Analysis Good Research Practices Task Force.* Value Health, 2016. **19**(4): p. 300-15.
21. Lancsar, E., D.G. Fiebig, and A.R. Hole, *Discrete Choice Experiments: A Guide to Model Specification, Estimation and Software.* Pharmacoeconomics, 2017. **35**(7): p. 697-716.
22. Hollin, I.L., et al., *Reporting Formative Qualitative Research to Support the Development of Quantitative Preference Study Protocols and Corresponding Survey Instruments: Guidelines for Authors and Reviewers.* Patient, 2020. **13**(1): p. 121-136.
23. Sinha, I.P., et al., *Development of a core outcome set for clinical trials in childhood asthma: a survey of clinicians, parents, and young people.* Trials, 2012. **13**: p. 103.
24. Fink, E.L., et al., *Development of a core outcome set for pediatric critical care outcomes research.* Contemp Clin Trials, 2020. **91**: p. 105968.
25. Allin, B., et al., *Outcomes at one-year post anastomosis from a national cohort of infants with oesophageal atresia.* PLoS One, 2014. **9**(8): p. e106149.
26. Allin, B., et al., *A UK wide cohort study describing management and outcomes for infants with surgical Necrotising Enterocolitis.* Sci Rep, 2017. **7**: p. 41149.
27. Allin, B.S.R., et al., *One-year outcomes following surgery for necrotising enterocolitis: a UK-wide cohort study.* Arch Dis Child Fetal Neonatal Ed, 2018. **103**(5): p. F461-F466.

For peer review only - http://bmjopen.bmj.com/site/about/guidelines.xhtml

28. Allin, B.S.R., et al., *Outcomes at five to eight years of age for children with Hirschsprung's disease.* Arch Dis Child, 2020: p. archdischild-2020-320310.
29. Allin, B.S.R., et al., *Management of Gastroschisis: Results From the NETS2G Study, a Joint British, Irish, and Canadian Prospective Cohort Study of 1268 Infants.* Ann Surg, 2021. **273**(6): p. 1207-1214.
30. Abellan-Perpinan, J.M., H. Bleichrodt, and J.L. Pinto-Prades, *The predictive validity of prospect theory versus expected utility in health utility measurement.* J Health Econ, 2009. **28**(6): p. 1039-47.
31. Long, A.M., et al., *Early population-based outcomes of infants born with congenital diaphragmatic hernia.* Arch Dis Child Fetal Neonatal Ed, 2018. **103**(6): p. F517-F522.
32. Long, A.M., et al., *Oesophageal atresia with no distal tracheoesophageal fistula: Management and outcomes from a population-based cohort.* J Pediatr Surg, 2017. **52**(2): p. 226-230.
33. Long, A.M., et al., *One-year outcomes of infants born with congenital diaphragmatic hernia: a national population cohort study.* Arch Dis Child Fetal Neonatal Ed, 2019. **104**(6): p. F643-F647.
34. Bradnock, T.J., et al., *Hirschsprung's disease in the UK and Ireland: incidence and anomalies.* Arch Dis Child, 2017. **102**(8): p. 722-727.
35. Bradnock, T.J., et al., *Gastroschisis: one year outcomes from national cohort study.* BMJ, 2011. **343**: p. d6749.
36. Brownlee, E., et al., *Current epidemiology and antenatal presentation of posterior urethral valves: Outcome of BAPS CASS National Audit.* J Pediatr Surg, 2019. **54**(2): p. 318-321.
37. Wragg, R., et al., *The postnatal management of boys in a national cohort of bladder outlet obstruction.* J Pediatr Surg, 2019. **54**(2): p. 313-317.
38. Wang, Y., et al., *One-year outcomes for congenital diaphragmatic hernia.* BJS Open, 2019. **3**(3): p. 305-313.
39. Ahmadi, S., et al., *Patient Preferences Around Extent of Surgery in Low-Risk Thyroid Cancer: A Discrete Choice Experiment.* Thyroid, 2020. **30**(7): p. 1044-1052.
40. van Dijk, J.D., et al., *An Empirical Comparison of Discrete Choice Experiment and Best-Worst Scaling to Estimate Stakeholders' Risk Tolerance for Hip Replacement Surgery.* Value Health, 2016. **19**(4): p. 316-22.
41. Szawlowski, S., et al., *How do surgeons' trade-off between patient outcomes and risk of complications in total knee arthroplasty? a discrete choice experiment in Australia.* BMJ Open, 2019. **9**(7): p. e029406.
42. Rowen, D., et al., *Review of Valuation Methods of Preference-Based Measures of Health for Economic Evaluation in Child and Adolescent Populations: Where are We Now and Where are We Going?* Pharmacoeconomics, 2020. **38**(4): p. 325-340.
43. Powell, P.A., et al., *Valuing child and adolescent health: a qualitative study on different perspectives and priorities taken by the adult general public.* Health Qual Life Outcomes, 2021. **19**(1): p. 222.
44. Craig, B.M., et al., *Valuation of Child Health-Related Quality of Life in the United States.* Health Econ, 2016. **25**(6): p. 768-77.
45. ChoiceMetrics, *Ngene 1.2 User Manual & Reference Guide*. 2018, Australia.
46. Allin, B.S.R., et al., *Development of a gastroschisis core outcome set.* Arch Dis Child Fetal Neonatal Ed, 2019. **104**(1): p. F76-f82.
47. Long, A.-M., *Short and long-term outcomes of children born with abdominal wall defects* in *National Perinatal Epidemiology Unit*. 2017, University of Oxford.
48. Reed Johnson, F., et al., *Constructing experimental designs for discrete-choice experiments: report of the ISPOR Conjoint Analysis Experimental Design Good Research Practices Task Force.* Value Health, 2013. **16**(1): p. 3-13.
49. Train, K., *Discrete choice methods with simulation*. 2nd ed. ed. 2009, Cambridge: New York, NY : Cambridge University Press.
50. Gonzalez, J.M., *A Guide to Measuring and Interpreting Attribute Importance.* Patient, 2019. **12**(3): p. 287-295.

For peer review only - http://bmjopen.bmj.com/site/about/guidelines.xhtml

Figure captions

Figure 1: A framework for stated-preference studies

For peer review only

For peer review only - http://bmjopen.bmj.com/site/about/guidelines.xhtml

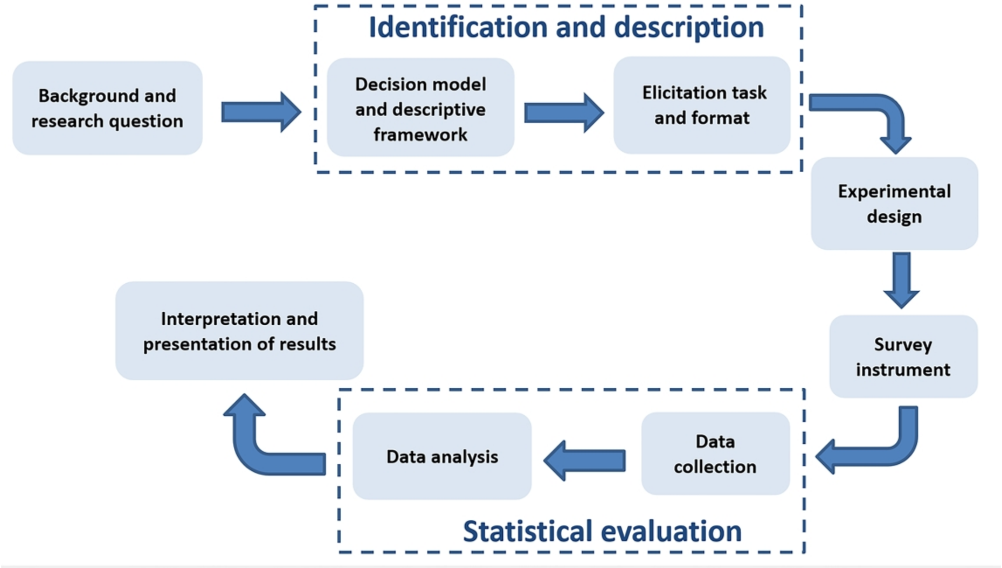

Figure 1: A framework for stated-preference studies

399x234mm (300 x 300 DPI)

Welcome and landing page

What is a successful treatment for a child with a surgical condition?
An online survey of people's preferences

Thank you for your interest in taking part in this survey.

Responses to the survey will help us improve the care of children who have a surgical condition.

If you are completing this survey using a tablet or smartphone, we recommend you use landscape mode.

In total, this survey will take about 15-20 minutes to complete. It will take approximately 10 minutes to work through the instructions and warm up questions, and about 5-10 minutes to complete the survey questions themselves.

Please click next to start.

Nuffield Department of POPULATION HEALTH

FUNDED BY NIHR | National Institute for Health Research

For peer review only - http://bmjopen.bmj.com/site/about/guidelines.xhtml

Screening questions

Screening question 1 of 3:

Please choose the option which *best* describes you:

- ◯ I am a parent or carer of a child who has been treated for a surgical condition
- ◯ I was treated as a child for a surgical condition
- ◯ I am a healthcare professional caring for children with surgical conditions

Screening question 2 of 3:

What is your age (years):

Screening question 3 of 3:

What is your gender?

- ◯ Female
- ◯ Male
- ◯ Other
- ◯ Prefer not to say

For peer review only

Background

Description of the characteristics used in the exercises

We would like you to consider scenarios describing what has happened to an imaginary child after they have been treated for a surgical condition. Before we show you the scenarios, we will take you through the *four characteristics* we will use to describe the scenarios in the exercises. You can read about the characteristics or watch short videos (less than one minute) describing them.

We will ask you some questions about each of these characteristics. **There are no right or wrong answers to these questions,** they are just to help you to get used to the characteristics.

Throughout the survey, the characteristics will be presented in the same order. However, this does not mean that one characteristic is more important than another.

Click next for the first *characteristic*.

Operations (Follow me for a short video)

Most surgical conditions are treated with one or more operations. Some operations that children undergo will be planned at the beginning of that child's treatment, whilst some will be emergencies. How complex an operation is also varies, from minor operations, such as draining an abscess (a pocket of pus under the skin), to more major operations, such as removing sections of intestine (bowel or gut).

In the scenarios you will be shown how many of each of the following types of operation an imaginary child undergoes:

- Planned major operations
- Planned minor operations
- Emergency major operations
- Emergency minor operations

We are only describing **the number of operations** the child had that are **related to their surgical condition**. For example, an operation for appendicitis would not be related.

Please answer the next questions about operations. **There are no right or wrong answers - we are just interested in what you think**.

How many major planned operations do you think are normally needed to treat a child with a broken leg?

No major planned operations	One major planned operation	Two major planned operations	Six major planned operations
○	○	○	○

How many minor planned operations do you think are normally needed to treat a child with a broken leg?

No minor planned operations	One minor planned operation	Two minor planned operations	Six minor planned operations
○	○	○	○

For peer review only - http://bmjopen.bmj.com/site/about/guidelines.xhtml

How many major emergency operations do you think are normally needed to treat a child with a broken leg?

No major emergency operations	One major emergency operation	Two major emergency operations	Six major emergency operations
○	○	○	○

How many minor emergency operations are normally needed to treat a child with a broken leg?

No minor emergency operations	One minor emergency operation	Two minor emergency operations	Six minor emergency operations
○	○	○	○

Infections treated in hospital (Follow me for a short video)

In some cases, an infection such as a collection of pus within the tummy, may occur shortly after an operation, whilst the child is still in hospital. In other cases, an infection may develop whilst the child is at home, but the infection is still related to their surgical condition and is serious enough for them to need treatment in hospital.

For the scenarios in this survey, you will be shown **the number of infections** the imaginary child had **that were related to their surgical condition and needed treatment in hospital**.

Please answer the next question about infections treated in hospital that will help you to get familar with the terminology used in this survey. **Please remember there are no right or wrong answers - we are just interested in what you think**.

How many infections treated in hospital do you think children usually develop after treatment for a broken leg?

No infections treated in hospital	One infection treated in hospital	Two infections treated in hospital	Six infections treated in hospital
○	○	○	○

For peer review only - http://bmjopen.bmj.com/site/about/guidelines.xhtml

Quality of life: The child's quality of life (Follow me for a short video)

In the scenarios we will describe the imaginary child's quality of life as **good, fair, or poor**.

Quality of life is very individual, and what one person thinks of as a good quality of life, someone else may describe as a fair quality of life.

Therefore, we would like you to decide for yourself what you think a good, fair, or poor quality of life for the imaginary child may be.

You might think about whether their condition has had any specific impact on them, for example, resulted in them being incontinent of wee or poo, or need for hospital care or visits. There may also be more broad factors to consider such as how much enjoyment a child gets from day-to-day life, the activities they can do for themselves, and impact on school or home life and family members.

Please read the short story about an imaginary child named Molly and answer the question that follows:

Molly is seven years old and several times a week has accidents where she passes poo in her underwear. Some children at school have started to bully her because of this. As a result, Molly doesn't enjoy school, and often tries to find reasons not to go. Over the last year her development has started to fall behind that of her peers. Molly gets quite stressed and anxious.

Which option below do you think best describes Molly's quality of life?

Good quality of life	Fair quality of life	Poor quality of life
○	○	○

Survival: How long the child survived after their diagnosis (Follow me for a short video)

In each of the scenarios we will describe **how long the imaginary child survived after they were diagnosed** with their surgical condition. This will be presented as one of the following categories:

- Lived more than twenty years, without any expectation that their surgical condition would shorten their life expectancy
- Lived twenty years
- Lived five years
- Lived one year
- Lived six months
- Lived one month

Please answer the next question about survival.

How long do you think children usually survive after being diagnosed with a broken leg?

Lived more than twenty years (normal life expectancy)	Lived twenty years	Lived five years	Lived one year	Lived six months	Lived one month
○	○	○	○	○	○

For peer review only - http://bmjopen.bmj.com/site/about/guidelines.xhtml

Practice question paired comparison

Exercise 1

In this exercise, you will be shown a pair of scenarios (**A** and **B**). In each scenario the four *characteristics* describe what has happened to an imaginary child with a surgical condition following their treatment.

We would like you to tell us **whether scenario A or scenario B describes the more successful result of the child's treatment.**

This exercise will be made up of 10 questions each presenting a pair of scenarios. **To make it easier to see the difference between the two scenarios, the characteristics that are the same in both scenarios have a grey background.**

Practice question for Exercise 1

On the next page you will see a practice question.

UNIVERSITY OF OXFORD

Which scenario describes the more successful result of the child's treatment?
Click Scenario A or B

Practice task:	Scenario A	Scenario B
Operations	Six major planned operations	No major planned operations
	One minor planned operation	One minor planned operation
	Six major emergency operations	No major emergency operations
	One minor emergency operation	One minor emergency operation
Infections treated in hospital	One infection treated in hospital	One infection treated in hospital
Quality of life	Poor quality of life	Good quality of life
Survival	Lived **one month**	Lived more than twenty years (normal life expectancy)
Click Scenario A or B	Scenario A	Scenario B

Exercise 1 practice question feedback

In the practice question you have just completed, you thought about the characteristics of both scenarios and decided that **Scenario B** was the more successful result of the child's treatment.

Please try to answer all of the following 10 questions by selecting the scenario you think is the **more successful result of the child's treatment.** Try to give each question equal attention.

Remember, there are no right or wrong answers – we are just interested in what you think.

For peer review only - http://bmjopen.bmj.com/site/about/guidelines.xhtml

Paired comparisons (participants to complete ten tasks)

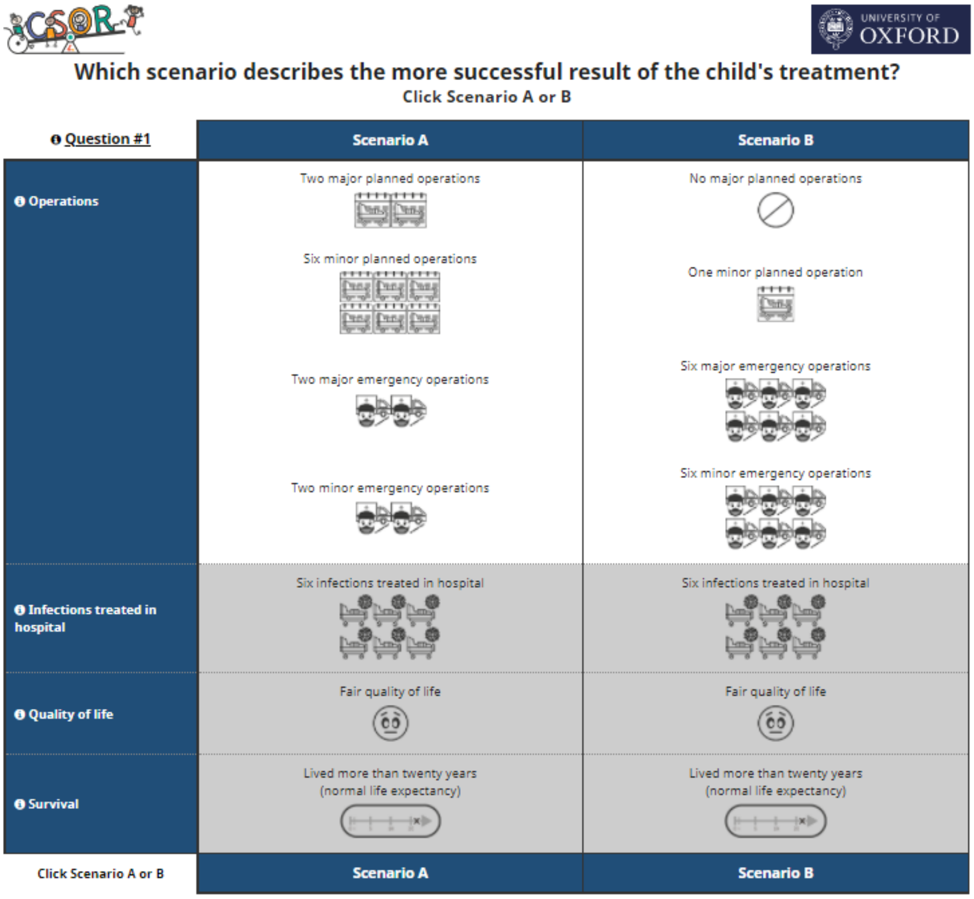

CSOR-T

UNIVERSITY OF OXFORD

Which scenario describes the more successful result of the child's treatment?
Click Scenario A or B

Question #1	Scenario A	Scenario B
Operations	Two major planned operations<br>Six minor planned operations<br>Two major emergency operations<br>Two minor emergency operations	No major planned operations<br>One minor planned operation<br>Six major emergency operations<br>Six minor emergency operations
Infections treated in hospital	Six infections treated in hospital	Six infections treated in hospital
Quality of life	Fair quality of life	Fair quality of life
Survival	Lived more than twenty years (normal life expectancy)	Lived more than twenty years (normal life expectancy)
Click Scenario A or B	Scenario A	Scenario B

NPEU

Nuffield Department of POPULATION HEALTH

FUNDED BY NIHR | National Institute for Health Research

For peer review only - http://bmjopen.bmj.com/site/about/guidelines.xhtml

Practice question kaizen task

Exercise 2

Each question in **Exercise 2** has three parts:

- In the **first** part you will select between two scenarios (**A** and **B**) as you did in Exercise 1.
- In the **second** part you will see one scenario, again described by the four characteristics. Next to some of the characteristics, you will see an alternative option. You will be asked to **choose three characteristics you think will improve the scenario, starting with the one you think will improve it the most**.
- In the **third** part you will select between two scenarios (**A** and **B**) as you did in Exercise 1.

Practice question for Exercise 2

On the next page you will see a practice question.

CSOR UNIVERSITY OF OXFORD

Which scenario describes the more successful result of the child's treatment?
Click Scenario A or B

Practice question: Part 1.	Scenario A	Scenario B
Operations	One major planned operation	No major planned operations
	One minor planned operation	No minor planned operations
	One major emergency operation	No major emergency operations
	One minor emergency operation	No minor emergency operations
Infections treated in hospital	One infection treated in hospital	No infections treated in hospital
Quality of life	Fair quality of life	Fair quality of life
Survival	Lived twenty years	Lived **one month**
Part 1. Click Scenario A or B	Scenario A	Scenario B

Which characteristics would you change to improve scenario A?
(Please choose three characteristics from the second column)

Practice task: Part 2.	Scenario A	Select the characteristics in the order you would change them
Operations	One major planned operation	
	One minor planned operation	No minor planned operations
	One major emergency operation	No major emergency operations
	One minor emergency operation	No minor emergency operations
Infections treated in hospital	One infection treated in hospital	No infections treated in hospital
Quality of life	Fair quality of life	
Survival	Lived twenty years	
Part 2.	Scenario A	Select the characteristics in the order you would change them

Clear Selection

For peer review only - http://bmjopen.bmj.com/site/about/guidelines.xhtml

Which scenario describes the more successful result of the child's treatment?

Click Scenario A or B

Practice Task: Part 3.	Scenario A	Scenario B
Operations	One major planned operation	No major planned operations
	No minor planned operations	No minor planned operations
	No major emergency operations	No major emergency operations
	No minor emergency operations	No minor emergency operations
Infections treated in hospital	No infections treated in hospital	No infections treated in hospital
Quality of life	Fair quality of life	Fair quality of life
Survival	Lived twenty years	Lived **one month**
Part 3. Click Scenario A or B	Scenario A	Scenario B

Exercise 2 practice question feedback

The practice question you have just completed had three parts:

- In the first part you selected **the Scenario A.**
- In the second part you improved three characteristics of **Scenario A:**
  - **First** from one minor planned operation to no minor planned operations
  - **Second** from one major emergency operation to no major emergency operations
  - **Third** from one minor emergency operation to no minor emergency operations
- In the third part you selected the improved **Scenario A.**

Please try to answer all of the following 3 questions. As in the practice question, in the **first part** of each question you need to select a scenario, in the **second part** you need to make three improvements starting with the one you think is most important, and in the **third part** you need to again select a scenario.

Remember, there are no right or wrong answers – we are just interested in what you think.

Kaizen tasks (participants to complete three tasks)

Which scenario describes the more successful result of the child's treatment?

Click Scenario A or B

Part 1.	Scenario A	Scenario B
Operations	Six major planned operations<br>One minor planned operation<br>Six major emergency operations<br>Two minor emergency operations	No major planned operations<br>No minor planned operations<br>No major emergency operations<br>No minor emergency operations
Infections treated in hospital	Six infections treated in hospital	No infections treated in hospital
Quality of life	Fair quality of life	Fair quality of life
Survival	Lived five years	Lived **one month**
Part 1. Click Scenario A or B	Scenario A	Scenario B

For peer review only - http://bmjopen.bmj.com/site/about/guidelines.xhtml

UNIVERSITY OF OXFORD

Which characteristics would you change to improve scenario A?

(Please choose three characteristics from the second column)

Question 2 Part 2.	Scenario A	Select the characteristics in the order you would change them
Operations	Six major planned operations	
	One minor planned operation	No minor planned operations
	Six major emergency operations	Two major emergency operations
	Two minor emergency operations	
Infections treated in hospital	Six infections treated in hospital	No infections treated in hospital
Quality of life	Fair quality of life	Good quality of life
Survival	Lived five years	
Part 2.	Scenario A	Select the characteristics in the order you would change them

Clear Selection

ew only

Which scenario describes the more successful result of the child's treatment?

Click Scenario A or B

Part 3.	Scenario A	Scenario B
Operations	Six major planned operations	No major planned operations
	No minor planned operations	No minor planned operations
	Two major emergency operations	No major emergency operations
	Two minor emergency operations	No minor emergency operations
Infections treated in hospital	No infections treated in hospital	No infections treated in hospital
Quality of life	Good quality of life	Fair quality of life
Survival	Lived five years	Lived one month
Part 3. Click Scenario A or B	Scenario A	Scenario B

For peer review only - http://bmjopen.bmj.com/site/about/guidelines.xhtml

Debriefing questions

Please tell us what you thought about the two different exercises you just answered

	Exercise 1: Choosing between scenario A and B	Leaning towards Exercise 1	About the same	Leaning towards Exercise 2	Exercise 2: Choosing between scenario A and B and select three improvements
Which exercise did you **prefer to complete**?	○	○	○	○	○
Which exercise did you find **easier to understand**?	○	○	○	○	○
Which exercise did you find **easier to complete**?	○	○	○	○	○

Would you like to comment about the two different exercises you just answered?

For peer review only

Demographics questions (parents and carers)

About you

To help us ensure the results are an accurate description of what a successful treatment is for a child with a surgical condition, it is important that we understand who has completed the survey. Please answer the following questions. It will not be possible to identify you from your responses.

Which country do you usually live in?

- UK
- Ireland
- Australia
- Canada
- New Zealand
- United States of America
- Other:

Please select the surgical condition(s) for which your child has been treated:

- Hirchsprung's disease
- Necrotising enterocolitis
- Gastroschisis
- Oesophageal atresia
- Congenital Diaphragmatic Hernia
- Posterior Urethral Valves
- Other:

Please select which age category your child falls into:

- My child has died
- Less than 1 year old
- 1 to 5 years old
- 6 to 10 years old
- 11 to 18 years old
- More than 18 years old

For peer review only - http://bmjopen.bmj.com/site/about/guidelines.xhtml

Please select how you would describe your child's current quality of life:

- Good quality of life
- Fair quality of life
- Poor quality of life
- Prefer not to answer

Are you:

- Single
- Married/Partner
- Separated
- Divorced
- Widowed
- Prefer not to answer

Please indicate which occupational group the main earner in your household belongs to, or which group fits best:

- Managers, directors and senior officials
- Professional/Associate Professional occupations
- Administrative and secretarial occupations
- Skilled trades occupations
- Caring, leisure and other service occupations
- Sales and customer service occupations
- Process, plant and machine operatives
- Elementary occupations
- Pensioner
- Unemployed
- Never employed
- Prefer not to answer

Do you have a Degree or equivalent professional qualification?

- Yes
- No

For peer review only - http://bmjopen.bmj.com/site/about/guidelines.xhtml

Have you experienced serious illness in addition to having a child with a surgical condition?

	Yes	No	Prefer not to answer
Yourself	○	○	○
In your family	○	○	○
As a carer for a family member	○	○	○

For peer review only

Demographics questions (individuals treated as child for surgical condition)

About you

To help us ensure the results are an accurate description of what a successful treatment is for a child with a surgical condition, it is important that we understand who has completed the survey. Please answer the following questions. It will not be possible to identify you from your responses.

Which country do you usually live in?

- UK
- Ireland
- Australia
- Canada
- New Zealand
- United States of America
- Other:

Please select the surgical condition(s) for which you were treated as a child:

- Hirchsprung's disease
- Necrotising enterocolitis
- Gastroschisis
- Oesophageal atresia
- Congenital Diaphragmatic Hernia
- Posterior Urethral Valves
- Other:

Please indicate which occupational group the main earner in your household belongs to, or which group fits best:

- Managers, directors and senior officials
- Professional/Associate Professional occupations
- Administrative and secretarial occupations
- Skilled trades occupations
- Caring, leisure and other service occupations
- Sales and customer service occupations
- Process, plant and machine operatives
- Elementary occupations
- Pensioner
- Unemployed
- Never employed
- Prefer not to answer

For peer review only - http://bmjopen.bmj.com/site/about/guidelines.xhtml

Do you have a Degree or equivalent professional qualification?

- Yes
- No

Have you experienced serious illness in addition to having a child with a surgical condition?

	Yes	No	Prefer not to answer
Yourself	○	○	○
In your family	○	○	○
As a carer for a family member	○	○	○

peer review only

For peer review only - http://bmjopen.bmj.com/site/about/guidelines.xhtml

Demographics questions (healthcare professionals)

About you

To help us ensure the results are an accurate description of what a successful treatment is for a child with a surgical condition, it is important that we understand who has completed the survey. Please answer the following questions. It will not be possible to identify you from your responses.

Which country do you usually live in?

- UK
- Ireland
- Australia
- Canada
- New Zealand
- United States of America
- Other:

What is your role/specialism?

- Surgeon
- Anaesthetist
- Paediatrician
- Neonatologist
- Nurse
- Advanced Nurse Practitioner/Specialist Nurse
- Other:

For surgeon, anaesthetist, paediatrician and neonatologist

Please select your highest current level of training:

- Consultant or equivalent
- Registrar or equivalent
- Foundation or core trainee
- Prefer not to answer

For nurse, advanced nurse practitioner, specialist nurse

Please select your highest current level of training:

- Consultant
- Modern Matron or Chief Nurse
- Advanced Nurse/Nurse practitioner
- Nursing specialist or Senior Nurse
- Newly qualified nurse
- Prefer not to answer

For peer review only - http://bmjopen.bmj.com/site/about/guidelines.xhtml

Have you experienced serious illness in addition to having a child with a surgical condition?

	Yes	No	Prefer not to answer
Yourself	○	○	○
In your family	○	○	○
As a carer for a family member	○	○	○

For peer review only

For peer review only - http://bmjopen.bmj.com/site/about/guidelines.xhtml

End of survey and thanks

Thank you for taking part in our survey

If you know other people that you think may be interested in taking part, please tell them about our study so they can register here.

Please do not share your link to the survey with others, as this is unique to you.

If you would like receive email updates on this project, as well as other opportunities to support research in child health, please contact the team at csor@npeu.ox.ac.uk. Your information will be kept securely at the University of Oxford and will not be used for any other purpose or shared with others.

If you would like to talk to someone about how completing the survey has affected you, please get in touch with the CSOR team and we will try to put you in contact with one of the many charities that have been supporting this project.

For peer review only - http://bmjopen.bmj.com/site/about/guidelines.xhtml

Supplementary material 2: Experimental design for the pair comparison

		Scenario 1							Scenario 2						
Choice situation	Block	planned_ major	planned_ minor	emergency_ major	emergency_ minor	infection	qol	survival	planned_ major	planned_ minor	emergency_ major	emergency_ minor	infection	qol	survival
1	3	3	4	3	4	4	3	4	4	4	3	1	3	3	5
2	4	1	3	4	2	4	2	1	2	1	1	2	4	3	1
3	4	1	1	1	3	4	1	1	4	1	4	2	3	1	1
4	5	2	1	3	3	4	3	5	4	1	4	3	3	3	1
5	3	4	2	4	3	4	1	4	4	3	4	1	4	3	3
6	5	4	1	2	4	3	3	3	4	1	4	2	4	3	2
7	3	2	1	2	4	4	3	1	2	1	4	3	3	3	5
8	3	1	1	3	3	4	3	1	3	1	4	1	4	1	1
9	1	4	2	3	1	4	3	5	4	3	2	4	4	2	5
10	2	4	1	3	4	1	1	2	4	3	2	2	1	3	2
11	5	2	4	3	2	2	3	3	2	4	3	4	1	2	5
12	1	3	3	1	4	3	1	1	4	1	2	2	3	1	1
13	4	3	3	4	1	2	3	2	3	4	4	4	2	2	1
14	4	2	2	4	2	4	2	3	4	3	2	2	4	2	1
15	3	2	1	4	1	2	2	5	2	1	4	3	1	3	3
16	3	3	4	4	4	2	3	1	4	1	3	4	2	3	2
17	1	1	4	1	1	3	1	1	2	2	2	1	1	1	1
18	3	2	4	2	2	1	3	4	3	1	2	3	1	3	1
19	3	2	3	3	2	1	3	2	2	4	2	1	4	3	2
20	5	2	3	2	3	2	1	1	4	3	2	1	1	1	4
21	2	2	3	4	4	3	3	2	4	1	4	4	2	3	4
22	2	3	2	2	1	3	3	3	3	3	2	3	2	3	1
23	1	4	1	4	3	1	2	3	4	2	3	3	4	1	3
24	4	3	3	3	3	4	3	5	3	4	3	4	1	3	2
25	5	3	1	2	2	4	1	2	3	1	3	1	2	1	4
26	1	2	4	2	4	3	2	2	3	4	2	2	1	2	3

For peer review only

For peer review only - http://bmjopen.bmj.com/site/about/guidelines.xhtml

27	1	3	1	2	2	2	2	5	4	1	4	1	2	2	1
28	5	4	1	2	1	1	2	4	4	2	2	1	2	1	1
29	2	3	2	3	2	4	3	4	4	2	4	2	1	2	4
30	5	3	1	3	4	4	2	1	4	2	1	4	2	2	1
31	2	3	1	2	3	3	1	1	3	4	2	1	2	3	1
32	1	3	1	3	1	4	2	3	3	1	3	4	3	3	2
33	5	2	1	4	3	2	3	4	2	3	4	3	4	1	3
34	4	1	2	2	1	3	3	1	4	2	1	1	4	1	1
35	4	1	4	2	2	2	1	1	4	1	1	2	2	2	1
36	4	2	1	2	4	1	3	1	2	1	3	2	3	1	1
37	2	2	1	2	1	3	1	4	2	4	2	3	2	1	2
38	1	2	1	4	4	3	3	4	4	2	4	4	3	2	2
39	3	3	4	2	3	1	2	5	3	4	3	1	2	2	3
40	2	1	1	1	2	2	3	1	4	1	1	1	4	2	1
41	1	1	2	4	4	4	2	1	3	4	3	3	4	2	1
42	5	3	1	4	4	4	3	3	3	2	3	4	1	3	4
43	2	2	2	3	4	2	3	1	3	1	3	1	2	3	4
44	4	2	3	4	3	3	1	1	3	3	4	2	1	3	1
45	2	3	1	4	3	3	2	1	3	2	4	2	3	1	4

For peer review only

Definitions of attribute levels:
planned_major; planned_minor; emergency_major; emergency_minor coded as:
1 = No operations
2 = One operation
3 = Two operations
4 = Six operations

Infection coded as:
1 = No infection treated in hospital
2 = One infection treated in hospital
3 = Two infections treated in hospital
4 = Six infections treated in hospital

For peer review only - http://bmjopen.bmj.com/site/about/guidelines.xhtml

Quality of life coded as:
1 = Good quality of life
2 = Fair quality of life
3 = Poor quality of life

Survival coded as:
1 = Lived more than twenty years (normal life expectancy)
2 = Lived twenty years
3 = Lived five years
4 = Lived one year
5 = Lived six months

For peer review only - http://bmjopen.bmj.com/site/about/guidelines.xhtml
